# Targeting *Leishmania major* Antigens to Dendritic Cells In Vivo Induces Protective Immunity

**DOI:** 10.1371/journal.pone.0067453

**Published:** 2013-06-26

**Authors:** Ines Matos, Olga Mizenina, Ashira Lubkin, Ralph M. Steinman, Juliana Idoyaga

**Affiliations:** Laboratory of Cellular Physiology and Immunology and Chris Browne Center for Immunology and Immune Diseases, The Rockefeller University, New York, New York, United States of America; INRS - Institut Armand Frappier, Canada

## Abstract

Efficient vaccination against the parasite *Leishmania major*, the causative agent of human cutaneous leishmaniasis, requires development of type 1 T-helper (Th1) CD4^+^ T cell immunity. Because of their unique capacity to initiate and modulate immune responses, dendritic cells (DCs) are attractive targets for development of novel vaccines. In this study, for the first time, we investigated the capacity of a DC-targeted vaccine to induce protective responses against *L. major*. To this end, we genetically engineered the N-terminal portion of the stress-inducible 1 protein of *L. major* (LmSTI1a) into anti-DEC205/CD205 (DEC) monoclonal antibody (mAb) and thereby delivered the conjugated protein to DEC^+^ DCs *in situ* in the intact animal. Delivery of LmSTI1a to adjuvant-matured DCs increased the frequency of antigen-specific CD4^+^ T cells producing IFN-γ^+^, IL-2^+^, and TNF-α^+^ in two different strains of mice (C57BL/6 and Balb/c), while such responses were not observed with the same doses of a control Ig-LmSTI1a mAb without receptor affinity or with non-targeted LmSTI1a protein. Using a peptide library for LmSTI1a, we identified at least two distinct CD4^+^ T cell mimetopes in each MHC class II haplotype, consistent with the induction of broad immunity. When we compared T cell immune responses generated after targeting DCs with LmSTI1a or other *L. major* antigens, including LACK (*Leishmania* receptor for activated C kinase) and LeIF (*Leishmania* eukaryotic ribosomal elongation and initiation factor 4a), we found that LmSTI1a was superior for generation of IFN-γ-producing CD4^+^ T cells, which correlated with higher protection of susceptible Balb/c mice to a challenge with *L. major*. For the first time, this study demonstrates the potential of a DC-targeted vaccine as a novel approach for cutaneous leishmaniasis, an increasing public health concern that has no currently available effective treatment.

## Introduction

Leishmaniases are a spectrum of diseases caused by different species of *Leishmania* spp. parasites, with clinical presentation ranging from a fatal visceral form (*L. donovani*) to a localized self-healing cutaneous lesion (*L. major*) [1]. In humans, acquired resistance to *L. major* infection is primarily mediated by cellular immunity, particularly antigen-specific Th1 CD4^+^ T cells [2]. Similarly, Th1-dependent protection is observed in mouse experimental models of *L. major* infection [3]. Resistant strains, such as C57BL/6, develop Th1 immune responses producing high levels of gamma interferon (IFN-γ), resulting in self-healing [3], [4], [5]. In contrast, Balb/c mice develop a typical Th2 response producing high amounts of IL-4, which is accompanied by disease progression after infection [6]. In susceptible Balb/c mice, protective Th1 T cell responses can be promoted by immunization [7], [8], [9], [10], [11], [12], suggesting that vaccines capable of generating potent and broad Th1 T cell responses can provide protective immunity to *Leishmania* infection. However, despite current evaluation of several strategies as potential candidates, there is no licensed vaccine available against *Leishmania* [13].

Dendritic cells (DCs) are highly specialized antigen-presenting cells essential for generation of protective T cell immune responses [14], [15]. Depending on the nature of the microbial stimulus, they can direct the development of polarized Th responses [16]. Thus, manipulation of the DC compartment for generation of antigen-specific Th1 T cell responses offers a promising strategy for vaccination against *Leishmania*. Accordingly, infusion of susceptible Balb/c mice with DCs loaded *ex vivo* with *L. major* antigens induces protective Th1 T cell responses [7], [17], [18], [19]. An alternative approach in the intact animal is the use of monoclonal antibodies (mAbs) against surface uptake receptors to deliver specific antigens to DCs *in situ*, within lymphoid tissues. Delivery of vaccine proteins within mAbs increases the efficiency of antigen presentation on MHC class II complexes by approximately 100-fold [20], [21], [22], [23], [24]. Importantly, DCs targeted with anti-receptor mAbs can induce protective Th1 CD4^+^ T cell responses when an appropriate adjuvant is coadministered [24], [25], [26]. In particular, we have shown that the synthetic form of viral double-stranded RNA, polyinosinic:polycytidylic acid (poly IC), and its more RNase-resistant analog stabilized with poly-L-lysine, poly ICLC, are superior adjuvants for generation of Th1 T cell responses induced by DC-targeted vaccines [26]. Hence, delivery of viral or bacterial antigens to DCs using fusion mAbs against DEC205/CD205 (DEC) coadministered with poly IC or poly ICLC results in Th1 CD4^+^ T cell responses that protect mice against recombinant vaccinia virus expressing gag-p24 or the bacterium *Yersinia pestis*, respectively [22], [25], [27], [28].

In the present study, we, for the first time, examined the potential of targeting DCs *in vivo* for induction of protective Th1 T cell immune responses against the parasite *L. major*. We initially focused our DC-targeting approach to the stress-inducible 1 antigen, an intracellular protein of *L. major* (LmSTl1) [29]. Evidence suggesting LmSTI1 is a good candidate for a protective vaccine includes the following: First, LmSTI1-specific Th1 T cells are found in draining lymph nodes of *L. major*-infected Balb/c mice [30]; second, high doses of soluble LmSTI1 protein or LmSTI1-encoding DNA, coadministered with IL-12, induce protection against *L. major* infection [31]; and third, Leish-111f (or LEISH-F1), a single recombinant poly-protein containing LmSTI1, induces Th1 T cell responses when administered with monophosphoryl lipid A (MPL) [32], [33] and has been recently shown to be safe and well tolerated in human subjects [34]. Our results demonstrated that delivery of the N-terminal domain of LmSTI1 to DCs in combination with DC maturation stimuli induced potent and broad antigen-specific CD4^+^ T cell responses and was able to protect susceptible Balb/c mice against a subsequent challenge with *L. major*. When we compared immune responses and protection induced by targeting LmSTI1 to DCs with the delivery of other *Leishmania* antigens, including LACK and LeIF, we found that LmSTI1a was superior for generation of IFN-γ-producing CD4^+^ T cells, which correlated with higher protection against a *L. major* challenge. Taken together, our study describes a novel strategy to induce consistent and highly effective immunity to the intracellular pathogen *L. major* and thus provides a promising new tool for a DC-based vaccine.

## Results

### LmSTI1, an Antigenic Protein Conserved between Species of *Leishmania*, can be Introduced into Anti-mouse DEC mAb

Th1 immune responses play a critical role in controlling leishmaniasis, and thus, antigens presented to T cells in MHC class II complexes have been considered good candidates for vaccines. An ideal vaccine antigen against *Leishmania* is expected to be conserved across different parasite species. Accordingly, the amino acid sequence of STI1 from *L. major* (LmSTI1) is >90% conserved with the STI1 sequence in *L. braziliensis*, *L. infantum*, and *L. donovani* (Figure S1), causative agents of mucocutaneous or visceral leishmaniasis, respectively. Furthermore, LmSTI1 lacks homology with mammalian proteins (not shown), which is desirable for a vaccine antigen to prevent unwanted autoimmune responses.

The LmSTI1 protein was initially cloned in frame into the heavy chain of anti-mouse DEC mAb; however, it was highly unstable and poorly expressed. Therefore, LmSTI1 was cleaved using an internal NotI site to yield a larger N-terminal portion (aa 1–398, LmSTI1a) and a smaller C-terminal portion (aa 401–546, LmSTI1b) (Figure S2A), which were both cloned in frame into anti-mouse DEC mAb and a control Ig mAb that has no receptor affinity (Figure S2B). The fusion mAbs were successfully expressed in 293T cells and purified in protein G columns. Because of the insertion of LmSTI1a or LmSTI1b, the heavy chain of the fused mAb was approximately 100 or 70 kDa, respectively, as shown by Coomassie blue staining (Figure S2C) and Western blotting (Figure S2D). Importantly, fusion of LmSTI1a or LmSTI1b into anti-DEC mAbs did not disrupt antibody function, as both anti-DEC-LmSTI1a and anti-DEC-LmSTI1b mAb efficiently bound to their corresponding receptor on stably transfected CHO cells but not to nontransfected CHO NEO cells (Figure S2E). Thus, anti-DEC mAb can be successfully engineered to express the LmSTI1 antigen from *L. major*. For our initial characterization of immune responses, LmSTI1a (N-terminal domain) was used, given that it covers approximately 72% of the native protein.

### Anti-DEC-LmSTI1a mAb Administered with a DC Maturation Stimulus Induces Multifunctional Th1 CD4^+^ T cells

**Figure 1:**
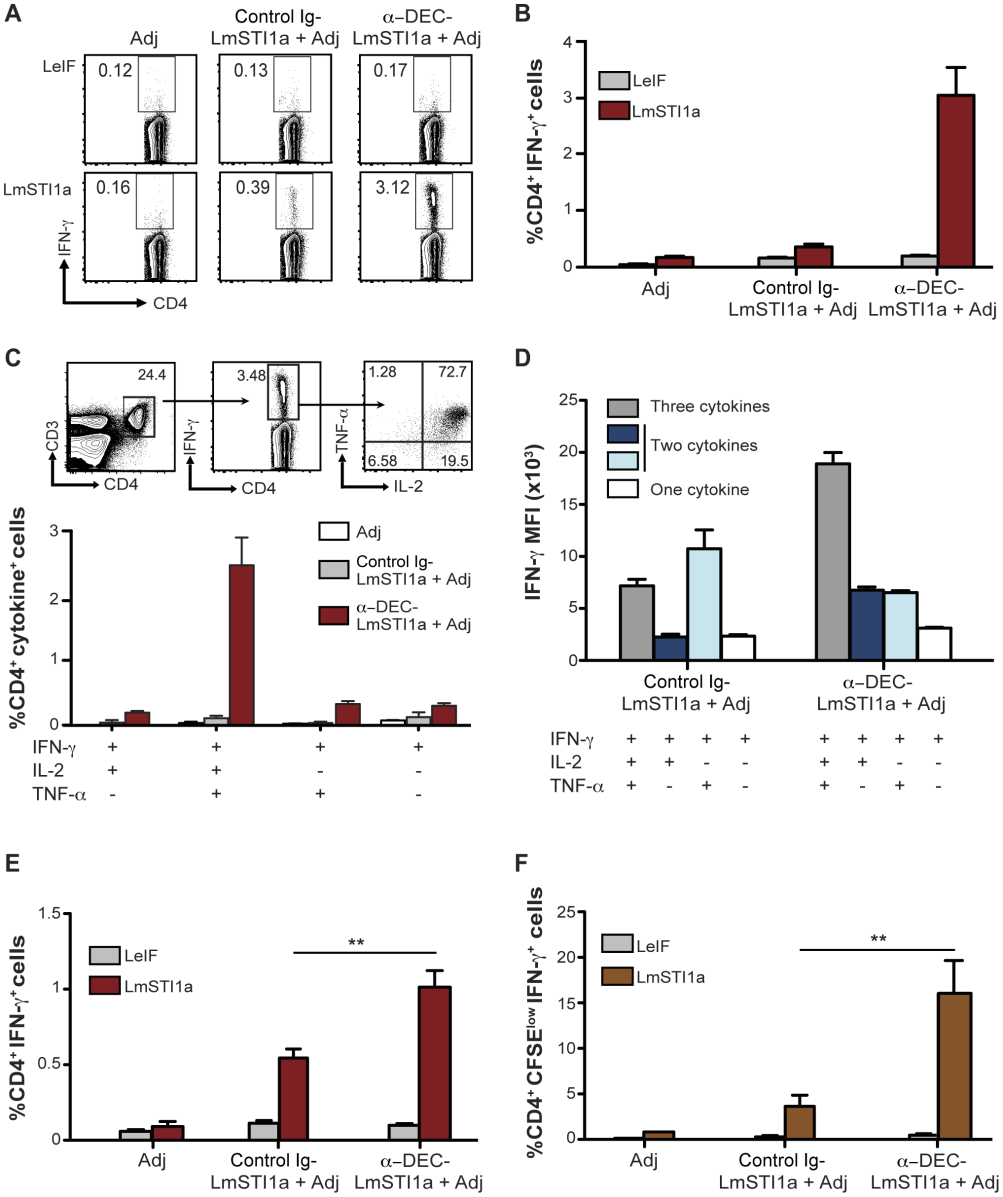
Single dose of anti-DEC-LmSTI1a immunizes multifunctional Th1 CD4^+^ T cells *in vivo*. (A) C57BL/6 mice were intraperitoneally inoculated with 1 µg of anti-DEC-LmSTI1a or control Ig-LmSTI1a plus 50 µg poly ICLC and 25 µg anti-CD40 mAb (Adjuvants, Adj). Fourteen days later, splenocytes were restimulated *in vitro* with LmSTI1a reactive peptide mix or LeIF nonreactive peptide mix in the presence of BFA. Intracellular cytokine staining was performed to detect IFN-γ in CD3^+^ CD4^+^ T cells. (B) As in A, but % of IFN-γ^+^CD4^+^ T cells is shown as mean ± SEM (n = 9). (C) Top panel shows the gating strategy to identify multifunctional T cells using multiparameter flow cytometry. CD3^+^ CD4^+^ IFN-γ^+^T cells (middle FACS plot) were analyzed for production of IL-2 and TNF-α (right FACS plot). Bottom bar graph shows the frequencies of total CD4^+^ T cells expressing each of the four cytokine combinations. Graph is expressed as the frequency of CD4^+^ CD3^+^ T cells and the mean ± SEM (n = 9). (D) IFN-γ median fluorescence intensity (MFI) of antigen-specific CD4^+^ T cells producing three, two, or one cytokines (IFN-γ alone or with TNF-α and/or IL-2) following immunization of C57BL/6 mice as in A. (E) As in A, but in Balb/c mice. The % of CD4^+^ T cells is shown as the mean ± SEM (n = 10). (F) As in E, but bulk splenocytes were CFSE-labeled and stimulated with LmSTI1a reactive peptide mix or LeIF nonreactive peptide mix for 4 days, wherein the cells were restimulated with LmSTI1a reactive peptide mix in the presence of BFA to detect IFN-γ^+^ cells in proliferating CFSE^low^ CD3^+^ CD4^+^ T cells. The frequency of CFSE^low^ IFN-γ^+^ CD4^+^ T cells is shown as the mean ± SEM (n = 6).

To determine T cell responses induced by LmSTI1a targeted to DCs via anti-DEC mAb, we inoculated C57BL/6 mice (H-2^b^) with 1 µg of mAb along with 50 µg poly ICLC and 25 µg anti-CD40 mAb (Adjuvant, Adj) as a DC maturation stimulus [22], [24]. As a control, we used Ig-LmSTI1a mAb with no receptor affinity. Two weeks after immunization, antigen-specific immune responses were evaluated by measuring IFN-γ-production in response to a reactive LmSTI1a peptide mix by multicolor flow cytometry. As shown in Figure 1A and quantified in Figure 1B, delivery of LmSTI1a within DEC mAb induced IFN-γ-producing CD4^+^ T cells. The frequency of the IFN-γ^+^ CD4^+^ T cells was greater when LmSTI1a was delivered using anti-DEC mAb compared with control Ig-LmSTI1a mAbs (Figure 1, A and B). These CD4^+^ T cells were antigen-specific since they only produced IFN-γ in response to LmSTI1a reactive peptide mix but not in response to a negative control LeIF peptide mix (Figure 1, A and B).

Previous studies revealed that protection against *L. major* infection in mice [35] and healing of cutaneous leishmaniasis in humans [36] correlates with the priming of multifunctional Th1 CD4^+^ T cells that simultaneously secrete high amounts of IFN-γ, IL-2, and TNF-α. Thus, we examined the capacity of LmSTI1a-specific IFN-γ^+^ CD4^+^ T cells to produce other cytokines. Following immunization with anti-DEC-LmSTI1a mAb, approximately 70% of the IFN-γ-producing CD4^+^ T cells, accounting for approximately 2–3% of the total CD4^+^ cells, also produced IL-2 and TNF-α (Figure 1C). Furthermore, CD4^+^ T cells producing three cytokines, i.e., IFN-γ, IL-2, and TNF-α, had the highest median fluorescence intensity (MFI) for IFN-γ compared with T cells producing only 1–2 cytokine (Figure 1D). This last parameter has also been associated with protective immunity to *Leishmania* infection [35], [36].

We then performed similar experiments in susceptible Balb/c (H-2^d^) mice, which typically develop a Th2 response to *L. major* infection. Anti-DEC-LmsTI1a mAb induced significantly higher percentage of antigen-specific IFN-γ-producing CD4^+^ T cells than control Ig-LmSTI1a mAb (Figure 1E), and the majority of these IFN-γ^+^ CD4^+^ T cells also produced TNF-α and IL-2 (Figure S3). Moreover, CD4^+^ T cells from mice immunized with anti-DEC-LmSTI1a mAb proliferated vigorously *in vitro* in response to the reactive LmSTI1a peptide mix and were able to produce higher amounts of IFN-γ after a second restimulation (Figure 1F).

Overall, these results show that delivery of LmSTI1a via anti-DEC mAb is effective at inducing significantly higher frequencies of antigen-specific CD4^+^ T cells producing elevated levels of three Th1 cytokines in two different MHC II haplotypes, H-2^b^ and H-2^d^. These features, i.e., multifunctionality, high cytokine production, and proliferation abilities, have been associated with generation of protective T cell immunity to cutaneous leishmaniasis.

### Targeting LmSTI1a to DCs via anti-DEC mAb is more Efficient than Non-targeted LmSTI1a Protein for Induction of Th1 CD4^+^ T cell Responses

**Figure 2:**
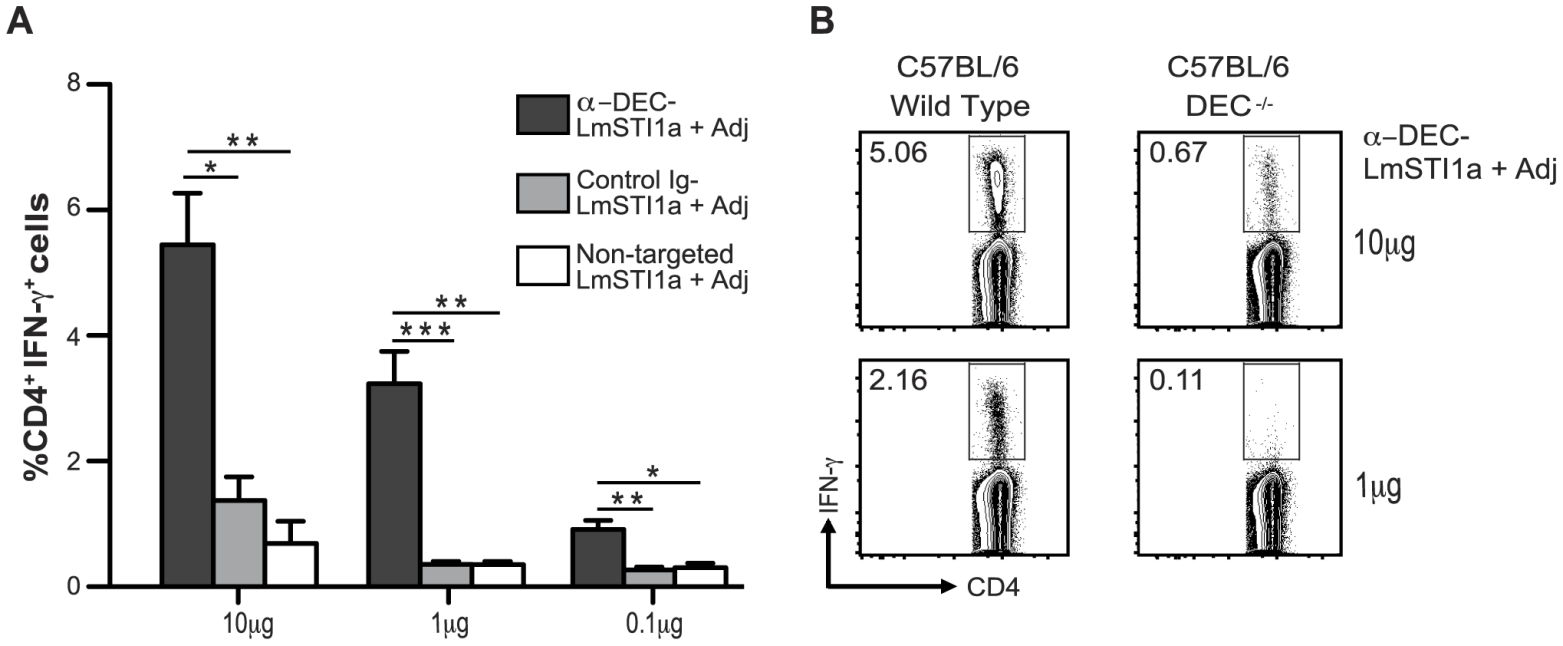
Priming of Th1 CD4^+^ T cells with anti-DEC-LmSTI1a *in vivo* requires targeting to DEC^+^ DCs. (A) C57BL/6 mice were intraperitoneally immunized with different doses of anti-DEC-LmSTI1a, control Ig-LmSTI1a, or non-targeted LmSTI1a protein (0.1–10 µg) in the presence of 50 µg poly ICLC and 25 µg anti-CD40 (Adj). Fourteen days after immunization, splenocytes were restimulated *in vitro* with a reactive LmSTI1a peptide mix for 6 h in the presence of BFA. Intracellular staining was performed to detect IFN-γ production in CD3^+^ CD4^+^ T cells. The mean ± SEM (n = 6). (B) As in A, but C57BL/6 wild-type or DEC^−/−^ mice were immunized with 10 or 1 µg of anti-DEC-LmSTI1a mAb plus Adj. Plots are representative of two experiments.

To evaluate the role of DCs in immunization, C57BL/6 mice were inoculated with graded doses of anti-DEC-LmSTI1a mAbs, control Ig-LmSTI1a mAb, or non-targeted LmSTI1a protein in the presence of the adjuvant. Anti-DEC-LmSTI1a was more efficient than control Ig-LmSTI1a at priming IFN-γ-producing CD4^+^ T cells and elicited significantly greater T cell responses than equivalent amounts of non-targeted protein (Figure 2A).

We further confirmed that these responses were receptor mediated. As shown in Figure 2B, T cell responses induced by anti-DEC-LmSTI1a in DEC-deficient mice were reduced approximately 10-fold (Figure 2B). Thus, DEC-mediated delivery of LmSTI1a antigen to DCs *in vivo* results in antigen-specific CD4^+^ T cell responses that are dependent on the presence of the DEC receptor.

### CD4^+^ T cell Responses Induced by anti-DEC-LmSTI1a are Directed to Several LmSTI1a Epitopes

**Figure 3:**
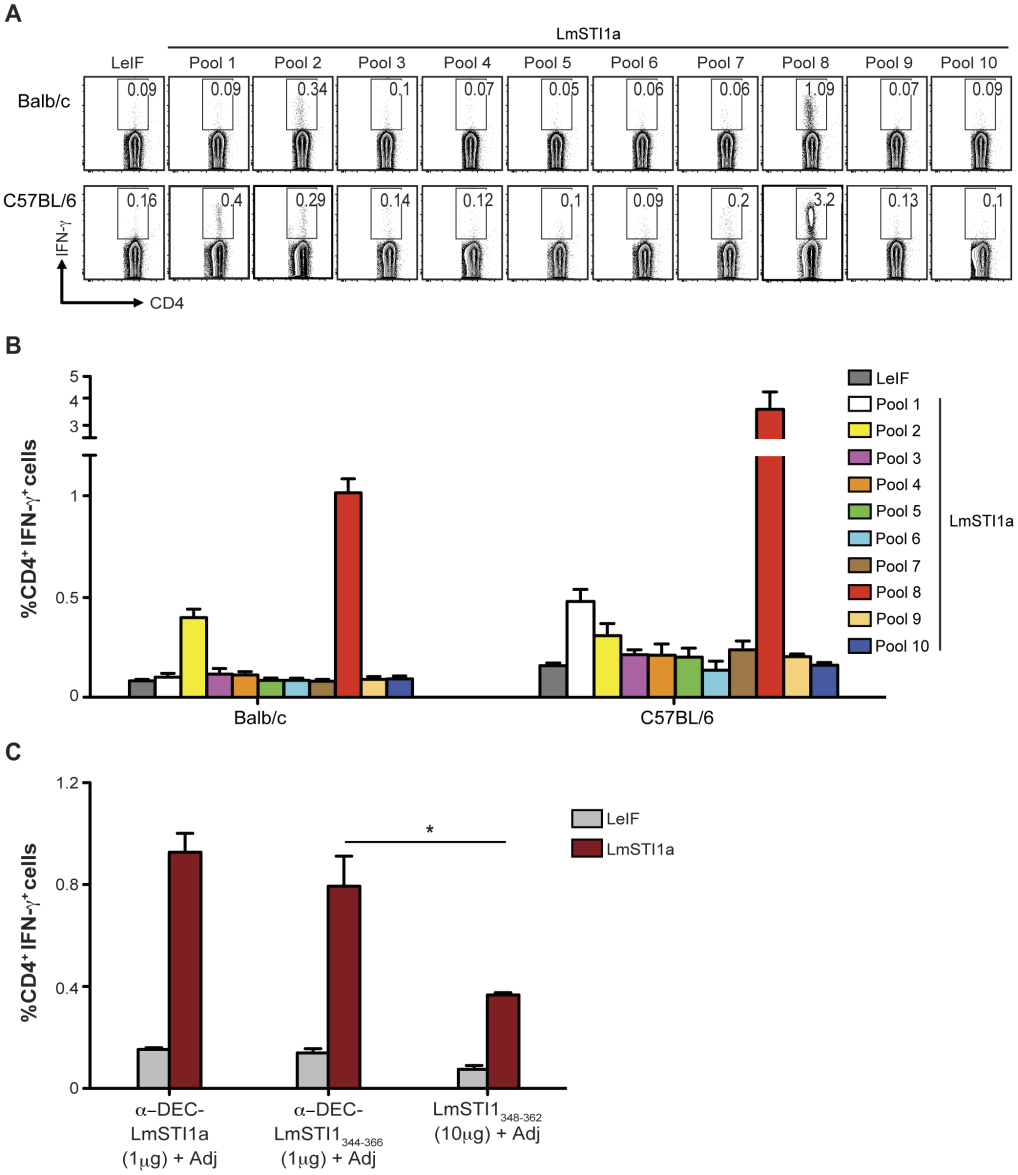
CD4^+^ T cell responses induced by anti-DEC-LmSTI1a are directed to several epitopes. (A) Balb/c or C57BL/6 mice were intraperitoneally immunized with 1 µg of anti-DEC-LmSTI1a plus poly ICLC (50 µg) and anti-CD40 mAb (25 µg) (Adj). Fourteen days later, splenocytes were restimulated with 10 different LmSTI1a peptide pools (2 µg/ml), each containing 12 individual peptides. IFN-γ-producing CD4^+^ T cells were assessed by intracellular cytokine staining. (B) As in A, but the percentage of IFN-γ^+^ CD4^+^ T cells is shown as mean ± SEM (n = 6). (C) Balb/c mice were immunized with 1 µg of anti-DEC-LmSTI1a or anti-DEC-LmSTI1_344–366_ mAbs or 10 µg of non-targeted LmSTI1_348–362_ immunodominant peptide in the presence of 50 µg poly ICLC and 25 µg anti-CD40 mAb (Adj). Production of IFN-γ was evaluated by FACS after restimulation with LmSTI1a reactive peptide mix or LeIF nonreactive peptide mix. The % of IFN-γ^+^ CD4^+^ T cells is shown as mean ± SEM (n = 3).

The breadth of antigen-specific T cell responses is reflected by the diversity of reactive epitopes recognized by T cells on distinct MHC class II haplotypes. To assess the breadth of the Th1 response and identify the individual LmSTI1a epitopes recognized by antigen-specific CD4^+^ T cells following immunization with anti-DEC-LmSTI1a mAb, we used a peptide library of 15-mer “mimetopes” staggered by 4aa along the 1–398 aa sequence of LmSTI1a. The library was divided into 10 sequential peptide pools, each containing 12 peptides. Each pool was used to restimulate splenocytes from mice immunized with anti-DEC-LmSTI1a mAb, and IFN-γ production was evaluated by FACS. As shown in Figure 3, CD4^+^ T cells from Balb/c and C57BL/6 mice responded to at least two different peptide pools, pool 2 and 8, whereas C57BL/6 mice also responded to peptide(s) in pool 1 (Figure 3, A and B). The aa sequences covered by LmSTI1a peptide pools 1, 2, and 8 are shown in Table S1.

We then identified individual reactive peptides in the H-2^d^ haplotype. Each peptide within pools 2 and 8 was used to restimulate splenocytes from Balb/c mice immunized with anti-DEC-LmSTI1a (Figure S4A). We found that the CD4^+^ T cell responses in Balb/c mice were directed to two overlapping peptides from each pool, i.e., peptides 16–17 in pool 2 and 87–88 in pool 8 (Figure S4A and Table 1). The sequences within these peptides cover two epitopes previously described in the H-2^d^ haplotype (Figure S4A, underlined aa sequences) [37])_._

**Table 1:** Identification of putative LmSTI1a-specific MHC II restricted peptides.

Strain	MHC IIhaplotype	LmSTIpeptide pool	Responding15-mer peptide	Position (aa)	Sequence	%IFN-γ^+^ CD4^+^ T cells
C57Bl/6	H-2^b^	1	p5	17–31	GRYVEAVNYFSKAIQ	0.526±0.159
			p6	21–35	EAVNYFSKAIQLDEQ	0.266±0.120
C57Bl/6	H-2^b^	2	p16	61–75	DKCISIKPNWAKGYV	0.315±0.198
			p17	65–79	SIKPNWAKGYVRRGA	0.236±0.146
C57Bl/6	H-2^b^	8	p88	348–362	VEEAYIDPEIAKQKK	3.052±0.982
			p89	352–366	YIDPEIAKQKKDEGN	2.335±0.776
Balb/c	H-2^d^	2	p16	61–75	DKCISIKPNWAKGYV	0.390±0.103
			p17	65–79	SIKPNWAKGYVRRGA	0.215±0.035
Balb/c	H-2^d^	8	p87	344–358	HQKAVEEAYIDPEIA	0.805±0.173
			p88	348–362	VEEAYIDPEIAKQKK	1.012±0.366

To identify individual reactive CD4 epitopes in the H-2^b^ haplotype, which have not been reported previously, C57BL/6 splenocytes of mice immunized with anti-DEC-LmSTI1a were restimulated with single peptides from pools 1, 2, and 8 (Figure S4B). Six peptides (two overlapping peptides from each pool) were able to induce IFN-γ production by CD4^+^ T cells from anti-DEC-LmSTI1a immunized mice (Figure S4B and Table 1). The dominant epitope was located within aa 348–366 (VEEAYIDPEIAKQKKDEGN) of the LmSTI1 sequence (Table 1).

Since the CD4^+^ T cell responses were mainly directed to three immunodominant overlapping peptides localized in pool 8, peptides 87, 88, and 89, we genetically engineered their peptide sequence into the C-terminal domain of the heavy chain of anti-DEC mAb (anti-DEC-LmsTI1_344–366_; Figure S2). Balb/c mice were immunized with anti-DEC-LmsTI1_344_–_366_ plus adjuvant, and 14 days later, we evaluated IFN-γ-producing CD4^+^ T cells by FACS. Inoculation of 1 µg of anti-DEC-LmSTI1_344–366_ mAb generated around two times more IFN-γ-producing CD4^+^ T cells than 10 µg of non-targeted LmSTI1_344–366_ peptide, suggesting that peptide targeting to DCs was approximately 20 times more efficient (Figure 3C). The frequency of CD4^+^ T cells producing IFN-γ after immunization with anti-DEC-LmSTI1_344–366_ was slightly lower than, but not significantly different from, animals immunized with anti-DEC-LmSTI1a mAbs (Figure 3C).

Thus, a single inoculation of LmSTI1a targeted to DCs using anti-DEC mAb results in broad CD4^+^ T cell responses directed to at least two different peptides in H-2^d^ and H-2^b^ backgrounds, including a dominant epitope localized within aa 344–366. In addition, when the immunodominant LmSTI_344–366_ peptide is delivered using anti-DEC mAb, the efficiency of targeting is preserved.

### LmSTI1 is a Superior Antigen for Induction of Th1 Responses

Identification of protective antigens is a major goal for generation of new and improved vaccines against *L. major*. Accordingly, we compared LmSTI1a with other previously described antigens of *Leishmania* spp., LACK and LeIF, for their capacity to induce IFN-γ-producing CD4^+^ T cells and protect against subsequent challenge with *L. major*.

Since the immunomodulatory properties of LeIF are localized in the N-terminal domain (aa 1–226) [38], we genetically engineered anti-DEC mAb to express LeIF_1–226_ (Figure S2, F–I). We also used for comparison anti-DEC-LACK mAb [20], and the C-terminus domain of the LmSTI1 gene (anti-DEC-LmSTI1b, Figure S2, A–E).

**Figure 4:**
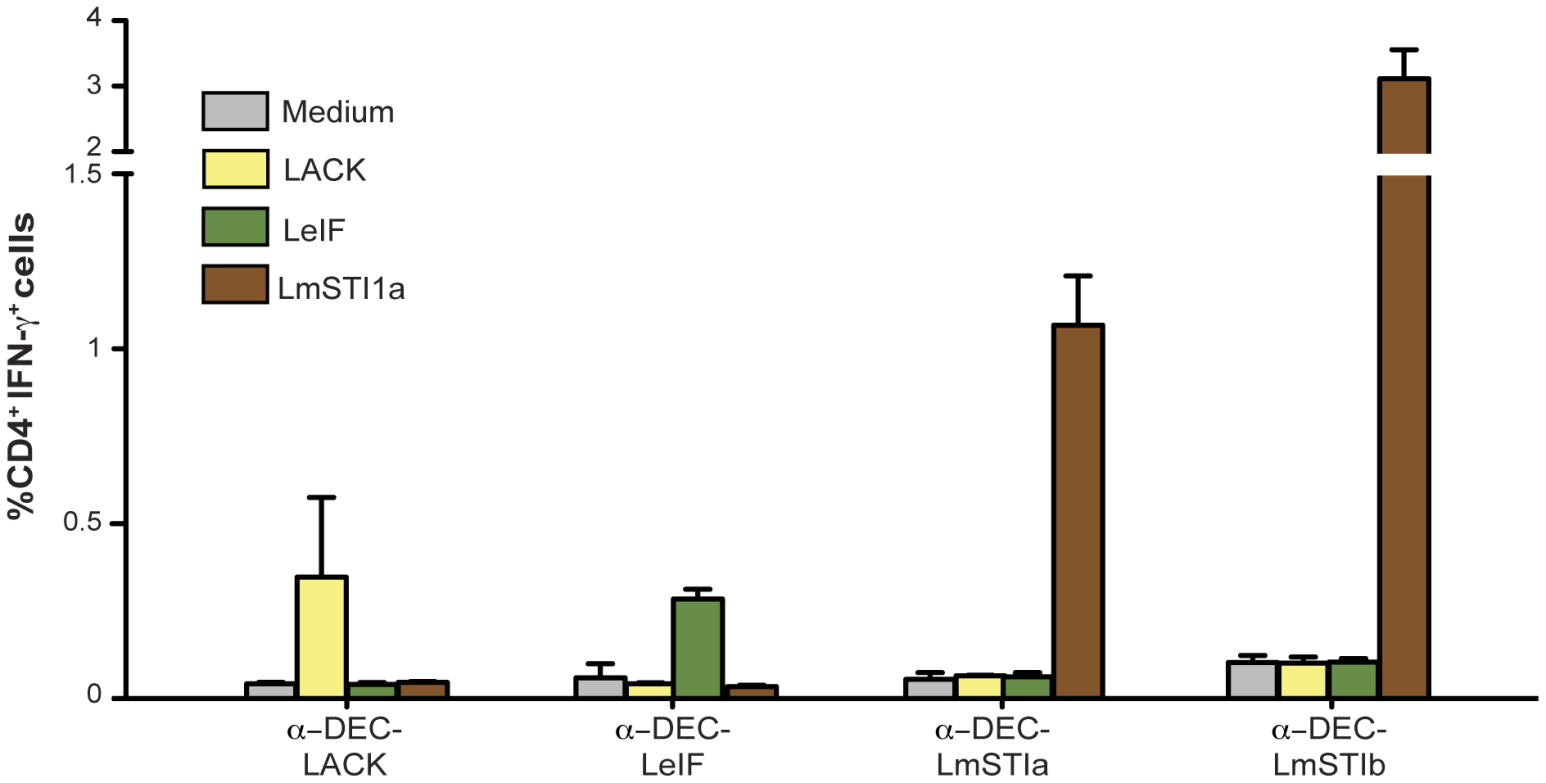
LmSTI1 targeted within anti-DEC mAbs induces superior Th1 immunity compared with other *L. major* antigens. Balb/c mice were intraperitoneally immunized with 10 µg of anti-DEC mAbs fused with LACK, LeIF, LmSTI1a, or LmSTI1b in the presence of 50 µg poly ICLC and 25 µg anti-CD40 mAb (Adj). Fourteen days after the immunization, splenocytes were restimulated *in vitro* with 2 µg of LACK, LeIF, or LmSTI1 peptide mix or medium alone (negative control) in the presence of BFA for 6 h. Intracellular cytokine staining was performed to detect IFN-γ^+^ cells on CD3^+^ CD4^+^ T cells. Data is shown as the mean ± SEM (n = 4–8).

Balb/c mice were immunized with anti-DEC mAb coupled with LmSTI1a, LmSTI1b, LACK, or LeIF along with the adjuvant, and antigen-specific responses were evaluated by detection of IFN-γ-producing CD4^+^ T cells. As shown in Figure 4, anti-DEC-LmSTI1a and anti-DEC-LmSTI1b induced the highest frequencies of IFN-γ^+^ CD4^+^ T cells compared with anti-DEC-LACK or LeIF. Epitope analysis revealed that the response to LACK was directed to a single, previously described epitope, corresponding to aa 156–173 (Table 2) [39]. The CD4^+^ T cell response to LeIF in Balb/c mice was also directed to a single, uncharacterized epitope localized in our pool 5 within aa 141–162 (Figure S4C and Table 2).

**Table 2:** Identification of LACK-, LeIF-, and LmSTI1b-specific CD4^+^ T cell responding peptides in Balb/c mice.

*L. major* antigen	Reactive peptide pool	Responding 15-mer peptide	Position (aa)	Sequence
LACK	1	p7–p8	156–173	ICFSPSLEHPIVVSGSWD 1
LeIF	5	p43–p45	141–162	LRKLQAGVIVAVGTPGRVSDVI
LmSTI1b	9–10	p107–p109	422–444	KPDFVKGYARKGHAYFWTKQYNR^2^

1 The sequence of the previously described reactive epitope in Balb/c mice is underlined [39].

2 The sequence of the previously described reactive epitope in Balb/c mice is underlined [37].

In contrast to targeting LACK or LeIF, delivery of LmSTI1b using anti-DEC mAb induced higher frequencies of CD4^+^ IFN-γ^+^ T cells than delivery of LmSTI1a (Figure 4). However, LmSTI1b-specific CD4^+^ T cell responses were directed to a single, previously described epitope [37] localized in our pools 9–10 (Figure S4D), within aa 422–444 (Table 2). Moreover, LmsTI1b, as well as LACK and LeIF, induced CD4^+^ T cell responses only in Balb/c mice and not in C57BL/6 mice (data not shown). Overall, we concluded that LmSTI1a has several advantages compared with other *L. major* antigens, including potent generation of Th1 T cell responses directed to at least two epitopes in two different haplotypes, i.e., H-2^b^ and H-2^d^.

### Targeting LmSTI1a to DCs Using mAbs against DEC Elicits Superior Protection against *L. major* Challenge

We next compared the protective potential of different *Leishmania* antigens delivered to DCs within mAb against DEC. To facilitate translation into the clinic, we inoculated fusion mAbs subcutaneously, which is one of the most attractive ways for vaccination in humans [40], [41], in a prime-boost regimen using poly ICLC as the adjuvant [24], [25].

**Figure 5:**
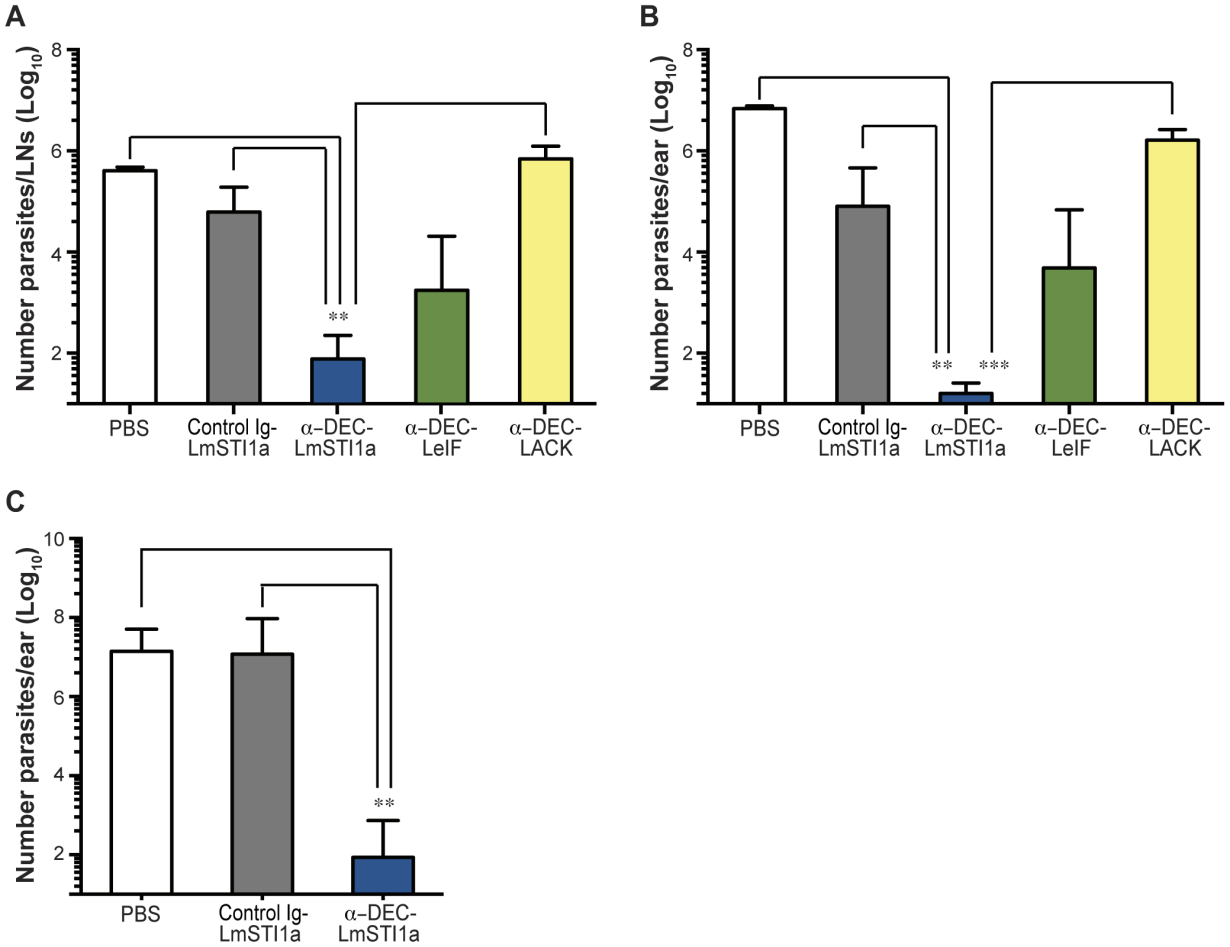
Anti-DEC-LmSTI1a mAb elicits protection against *L. major*. Balb/c mice were vaccinated in a prime-boost regimen consisting of two subcutaneous doses, administered 1 month apart, with 10 µg or 1 µg of control Ig-LmSTI1a or anti-DEC-LmSTI1a and 10 µg of anti-DEC-LACK or anti-DEC-LeIF mAbs in the presence of 50 µg poly ICLC. Ten to 15 days after boost, the mice were intradermally injected in the ear pinnae with 200–1000 *L. major* metacyclic promastigotes. Quantification of parasites obtained from ear-infected draining LN (A) or per infected ears (B) were calculated 12 weeks after challenge. Shown is the mean ± SEM (n = 5–12, except PBS, n = 2). (C) As in B, but Balb/c mice were intradermally challenged with 200–1000 *L. major* metacyclic promastigotes 12 weeks after the last immunization. Shown is the mean ± SEM (n = 5, except PBS, n = 3).

Susceptible Balb/c mice vaccinated with different antigens fused to anti-DEC mAbs were challenged 10–15 days later with a low dose of metacyclic promastigotes administered intradermally in the ear pinnae. Lesion development and parasite burden was evaluated in the challenged site and ear-draining lymph nodes (LNs). Vaccination with anti-DEC-LmSTI1a mAb plus poly ICLC resulted in approximately 100% protection (16 mice in four independent experiments), i.e., reduced pinnae edema and necrosis. In contrast, anti-DEC-LeIF mAb resulted in approximately 30–40% protection (six of 16 mice in four different experiments), while anti-DEC-LACK failed to induce protection against *L. major* infection (Figure S5A). Skin lesions were correlated with parasite numbers in the ear and ear-infected draining LNs (Figure 5, A and B). Mice vaccinated with anti-DEC-LmSTI1a mAb showed significantly lower number of parasites at the ear-infected draining LN (Figure 5A) compared with control-Ig LmSTIa and almost no parasites in the ear pinnae (Figure 5B). Vaccination with anti-DEC-LeIF mAb also exhibited moderate reduction in the size of the lesions, which correlated with a lower number of parasites in the LN and ear pinnea than that in PBS-treated animals, but was not as potent as treatment with anti-DEC-LmSTI1a (Figure 5, A and B). On the other hand, mice treated with anti-DEC-LACK or control Ig-LmSTIa mAbs showed the highest number of parasites in the infection site and the ear-infected draining LN (Figure 5, A and B).

The protective effect of anti-DEC-LmSTI1a mAb was also observed when Balb/c mice were challenged with a high dose of metacyclic promastigotes administered subcutaneously in the footpad (Figure S5B). Again, anti-DEC-LmSTI1a mAbs conferred complete protection (100% of the animals) with negligible footpad swelling and undetectable parasites in the lesions, whereas anti-DEC-LeIF and anti-DEC-LmSTI1b resulted in partial protection (40% and 80% of the animals, respectively; Figure S5B and C).

We further evaluated the protective effect of anti-DEC-LmSTI1a in the innately resistant mouse strain C57BL/6. As shown in Figure S5D, vaccination of C57BL/6 mice with anti-DEC-LmSTI1a mAb plus poly ICLC could confer some degree of protection against a low dose of metacyclic promastigotes administered intradermally represented by the reduced number of parasites detected in the infection site.

Finally, we evaluated the ability of anti-DEC-LmSTI1a to confer long-term protection against infection. Balb/c mice immunized with anti-DEC-LmSTI1a mAb plus adjuvant were challenged with live metacyclic promastigotes 12 weeks after the last immunization. Only immunization with anti-DEC-LmSTI1a, but not a control Ig-LmSTI1a mAb, could confer durable protection (100% of the animals), which correlated with a low number of parasites in the ears and ear-infected draining LN (Figure 5C, data now shown).

**Figure 6:**
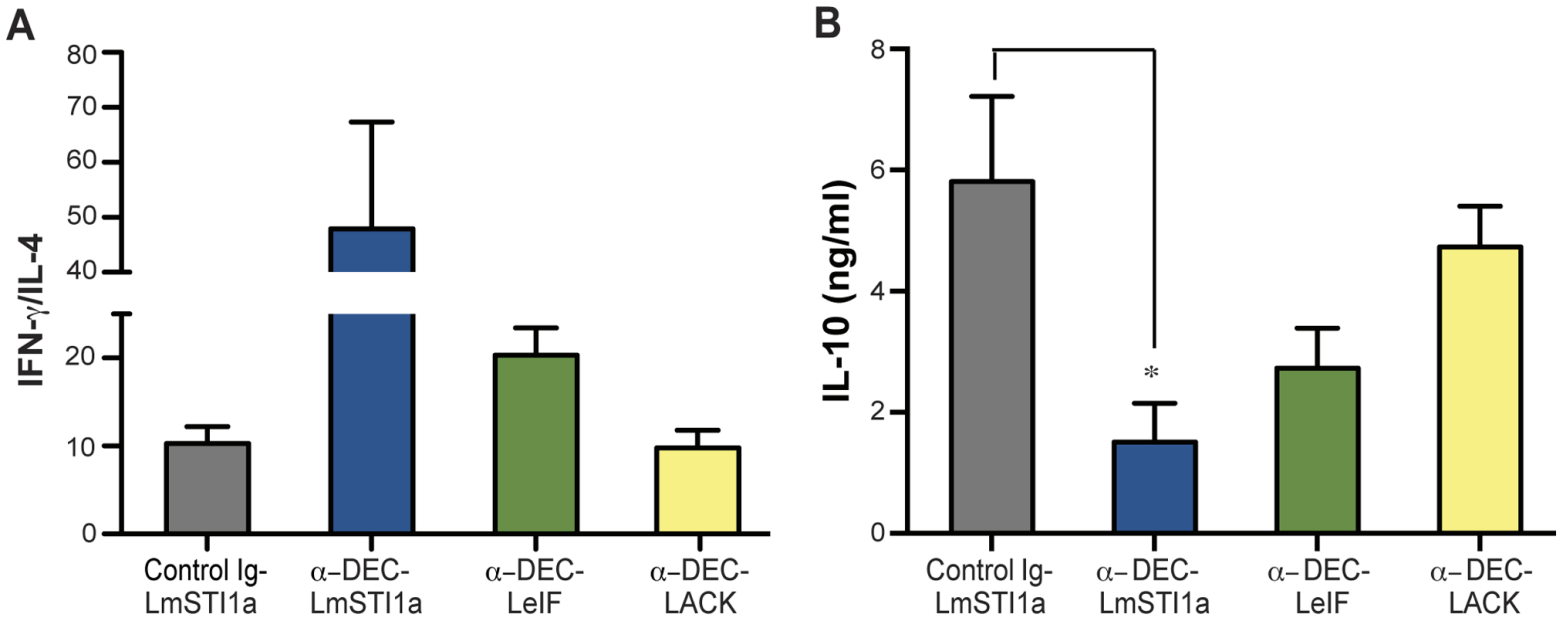
Analysis of the cytokine responses after challenge with *L. major*. As in Figure 5, but 12 weeks after infection with *L. major*, total cell suspensions of ear-infected draining LN were restimulated *in vitro* for 72 h with 10 µg/ml of SLA. The levels of IFN-γ, IL-4, and IL-10 cytokines in the culture supernatants were determined by ELISA. Data is shown as the mean ± SEM of the ratio of (A) IFN-γ/IL-4 and (B) IL-10 (n = 4–10).

To examine the nature of the protection induced by anti-DEC-LmSTI1a mAb, we analyzed IFN-γ, IL-4, and IL-10 responses 12 weeks after challenge with *L. major*. Lymphocytes from ear-infected draining LN from mice immunized with anti-DEC-LmSTI1a produced significantly less IL-4 in response to stimulation *ex vivo* with soluble *Leishmania* antigen (SLA) but comparable levels of IFN-γ compared with mice inoculated with control Ig-LmSTI1 (Figure S6, A and B). Consequently, the IFN-γ/IL-4 ratios in mice vaccinated with anti-DEC-LmSTI1a were highest compared with control Ig-LmSTI1, anti-DEC-LeIF, or anti-DEC-LACK (Figure 6A). Similarly, lymphocytes from mice vaccinated with anti-DEC-LmSTI1a produced significantly less IL-10 after stimulation *ex vivo* with SLA (Figure 6B). Taken together, these results suggest that the superior protection in anti-DEC-LmSTI1a vaccinated mice can be explained, at least in part, by a shift toward a Th1 phenotype.

Overall, we concluded that LmSTI1 targeted to DCs via DEC mAbs is the most effective *L. major* antigen to prevent cutaneous leishmaniasis.

## Discussion

Given the lack of effective and low-cost treatment, several strategies are being evaluated as potential vaccine candidates against leishmaniasis [7], [8], [9], [10], [11]; however, there are still no licensed vaccines [42]. Considering their unique capacity to induce and regulate immune responses, DCs offer a promising approach for development of novel vaccines. It has been previously shown that adoptive transfer of DCs loaded *ex vivo* with parasite antigens can mediate protection against *Leishmania* infection [7], [19], [43]. However, this approach relies on laborious and expensive isolation and *ex vivo* culture of cells. A novel approach is targeting antigens to DCs *in situ* in the intact animal using mAbs against DEC. Despite previous success using this DC-targeting approach for induction of protective immune responses against viruses [22], bacteria [27], [28], and even tumor cells [44], [45], to the best of our knowledge, this is the first report evaluating the efficacy of this strategy for generation of protective immune responses against a protozoan parasite. The findings in this report show that delivery of LmSTI1 to adjuvant-matured DCs was sufficient to induce protective immunity to *L. major*, one of the causative agents of human cutaneous leishmaniasis.

We chose LmSTI1 based on several criteria. STI1 is a protein highly conserved among different *Leishmania* species, suggesting that in addition to being useful for *L. major* infection, an anti-DEC-LmSTI1a vaccine may be useful for other forms of leishmaniasis, including visceral and mucosal leishmaniasis. LmSTI1 is present in the amastigote and promastigote form of the parasite [29]. Furthermore, LmSTI1 is divergent from its mammalian protein counterpart, indicating a decreased risk for undesirable pathological crossactivation of autoreactive T and B cells. Importantly, immunization with recombinant LmSTI1 protein administered alone or as a poly protein fused with other parasite proteins induces protection in susceptible Balb/c mice [32], [33], [46]. Our results demonstrate that anti-DEC-LmSTI1a induces several fold greater responses than LmSTI1 protein alone and confers a high level of protection against challenge with *L. major* in susceptible Balb/c and resistant C57BL/6 mice. Protection against *L. major* infection was consistent, even at low doses (1 µg) of fusion mAbs, and was durable for at least 12 weeks after immunization. In addition, anti-DEC-LmSTI1a protection was achieved despite increasing the parasite load (200–1×10^6^), or varying the site of infection (intradermal and subcutaneous).

Protection against *Leishmania* in experimental mouse models is thought to be primarily mediated by the production of IFN-γ [3]. The main source of IFN-γ is antigen-specific CD4^+^ T cells [47]. Furthermore, it was recently shown that vaccine-induced protection against *L. major* strongly correlates with generation of multifunctional Th1 CD4^+^ T cells producing high levels of IFN-γ, IL-2, and TNF-α [35]. Therefore, protection against infection seems to be dependent not only on the magnitude but also on the quality of the CD4^+^ T cell response generated. Accordingly, delivery of LmSTI1a to DCs via DEC mAbs in combination with a DC maturation stimulus induced potent multifunctional (IFN-γ^+^/IL-2^+^/TNF-α^+^) antigen-specific CD4^+^ T cells. Additionally, vaccine-induced LmSTI1a-specific CD4^+^ T cells proliferated vigorously and secreted high amounts of IFN-γ upon antigen restimulation *in vitro*.

The quality of the CD4^+^ T cell response induced by anti-DEC-LmSTI1a mAbs was also reflected by its breadth, i.e., T cell responses were produced in two different MHC haplotypes (H-2^b^ and H-2^d^), and the response was directed to at least two peptides in each haplotype. The peptides recognized following anti-DEC-LmSTI1a immunization in Balb/c mice were identical to those reported previously in H-2^d^ [37]. We extended the analysis to identify peptides recognized by CD4^+^ T cells in C57BL/6 (H-2^b^), which had not been assessed previously. Interestingly, the CD4^+^ T cell response was mainly directed to a dominant peptide, similar in sequence to the one recognized in Balb/c mice. Consequently, we delivered the LmSTI1_344–366_ peptide to DCs using anti-DEC mAbs, and as expected, this strategy also immunizes CD4^+^ T cells.

We further compared other *L. major* proteins as vaccine candidates by DEC targeting to DCs. LACK is another well-conserved *Leishmania* antigen; however, previous reports indicate that LACK fails to elicit long-term protective immune responses against *L. major* or other *Leishmania* species [48], [49]. Despite the ability of anti-DEC-LACK mAbs to prime some IFN-γ^+^ CD4^+^ T cells in agreement with a previous report [20], LACK targeting did not confer any protection against a low-dose *L. major* challenge in susceptible Balb/c mice, suggesting that this antigen is not adequate for traditional and/or DC-targeting immunization approaches.

On the other hand, LeIF antigen can expand antigen-specific Th1 cells [38], [50], and in our DEC DC-targeting model, it induced detectable levels of antigen-specific IFN-γ-producing CD4^+^ T cells. Distinct from LmSTI1a, responses induced by LeIF were restricted to H-2^d^, as shown by the lack of CD4^+^ T cell induction in C57BL/6 mice. Immunization of Balb/c mice with anti-DEC-LeIF induced partial protection (30–40%) following challenge with *L. major*. This could be related to the fact that the CD4^+^ T cell response with anti-DEC-LeIF was directed to only one epitope and induced lower frequencies of IFN-γ^+^ CD4^+^ T cells compared with LmSTI1a.

Given that the entire LmSTI1 protein could not be coupled to anti-DEC mAb, we performed a partial comparison of the N-terminal (LmSTI1a) and C-terminal (LmSTI1b) portions. Compared with LmSTI1a, LmSTI1b-specific CD4^+^ T cell responses were restricted to H-2^d^ and were directed to a single immunodominant epitope. Nevertheless, anti-DEC-LmSTI1b mAb induced high frequencies of IFN-γ^+^ CD4^+^ T cells and protective responses to a high-dose subcutaneous challenge with *L. major*. In the future, it will be interesting to study the effectiveness of a DC-based vaccine that includes more than one *Leishmania* antigen, e.g., LmSTI1a, LmSTI1b, and LeIF. Such a vaccine, Leish-111f [32], [51], is currently being studied with protective responses; however, it would be interesting to see if its efficacy is increased by DC targeting.

Despite the antigen being targeted, we found that protection against *L. major* infection was associated with a significant difference in the IFN-γ/IL-4 cytokine ratio, driven mainly by the decreased secretion of IL-4 in protected mice. Interestingly, we also found a decrease in the secretion of IL-10 in anti-DEC-LmSTI1a vaccinated mice. These results suggest that although additional, unexplored mechanisms may be involved, a Th1-biased response is correlated with protective immunity.

Efficient induction of CD4^+^ T cell responses in our model was dependent on the expression of the DEC receptor, as shown in DEC-deficient mice. In mice, DEC is expressed by a subset of DCs coexpressing CD8α that is able to produce high levels of IL-12 in response to microbial signals [52], [53]. This ability to produce IL-12 is critical for generation of Th1 responses. Indeed, targeting CD8αα^+^ DCs, but not CD8αα^−^ DCs, leads to a large polarized antigen-specific Th1 response [20], [24]. Furthermore, skin migratory DC subsets, including Langerhans cells and dermal DCs, express high levels of DEC and may be involved in generation of protective Th1-dependent immunity via IL-12 [3]. Further studies are needed to investigate the role of distinct DCs subsets in generation of protective Th1 T cell responses after targeting of leishmania antigens to DEC^+^ DCs.

Priming of Th1 CD4^+^ T cell responses using anti-DEC mAb requires maturation of DCs with TLR agonists. In this study, we selected poly ICLC, a microbial mimic TLR-3 and MDA5 agonist, because of its capacity to promote strong Th1 responses [24], [25], [26] and its superiority compared with other adjuvants, e.g., GLA-SE [54]. Importantly, poly ICLC is safe and well tolerated in humans [55].

Preventive vaccines are considered the best and most cost-effective approaches against pathogens, including *Leishmania*. Here we demonstrate how DCs can be exploited to prevent infection induced by the human intracellular pathogen *L. major*. As we learn more about DCs, we can decide about different targeting strategies. For example, we can direct antigens to distinct subsets of DCs *in vivo* using mAbs against uptake receptors differentially expressed by DC populations. In this regard, it would be interesting to evaluate targeting of *Leishmania* antigens to CD207/Langerin^+^ DC, including Langerhans cells, which are one of the parasite’s targets [56]. Furthermore, *Leishmania* antigens can be delivered to macrophages, the cell type most infected by the parasite [57], using mAbs against Treml4 [45].

Taken together, our results show that delivery of LmSTI1a to adjuvant-matured DCs significantly enhances antiparasite immunity. This strategy would seem logical to pursue clinically as a feasible approach for *Leishmania* infections.

## Materials and Methods

### Mice

We purchased female Balb/cJ and C57BL/6J mice from The Jackson Laboratory. DEC^−/−^ mice were kindly provided by M. Nussenzweig (The Rockefeller University, New York, NY) and bred in house. The mice were maintained under specific pathogen-free conditions and used at 6–8 weeks of age.

### Ethics Statement

This study was conducted in accordance with the recommendations in the Guide for the Care and Use of Laboratory Animals of the National Institutes of Health. The protocol was approved by Animal Care and Use Committee at The Rockefeller University (Protocol Number: 11414). All surgery was performed under sodium pentobarbital anesthesia, and all efforts were made to minimize suffering.

### Reagents

The following fluorescent conjugated mAbs were purchased from eBioscience (San Diego, CA) or BD Biosciences (Franklin Lakes, NJ): Alexa-488 anti-IL-2 (JES6-5H4), eFluor-450 or Alexa-700 anti-CD3 (500A2), Alexa-700 or PE anti-CD4 (RM4-5), APC or PE-Cy7 anti-IFN-γ (XMG1.2), and PE-Cy7 anti-TNF-α MP6-XT22). Other reagents were live/dead fixable aqua or violet vitality dye from Life Technologies (Grand Island, NY), and CFSE (5,6-carboxyfluorescein diacetate succinimidyl ester, Life Technologies). Overlapping (staggered by four amino acids) 15-mer peptides covering the LmSTI1, LeIF, or LACK sequences were synthesized by H. Zebroski in the Proteomics Resource Center of The Rockefeller University.

### Engineering and Production of Fusion mAbs and LmsTI1a Soluble Protein

Open reading frames were amplified by PCR from genomic DNA generously provided by Dr. Nicholas Glaichenhaus (Friedlin strain of *L. major*), with the sets of primers described in Table S2. DNA coding for LmSTI1_344–366_ peptide was generated by the annealing of complementary synthetic oligonucleotides (Table S2). The PCR products were inserted in frame to the C-terminus of the heavy chain of anti-mouse-DEC (NLDC; [58], [59], [60]), and a control Ig mAb with no receptor affinity (GL117; [60]). The fusion mAbs were expressed by transient transfection using calcium phosphate in 293T cells in the presence of serum-free DMEM medium supplemented with Nutridoma SP (Roche Applied Science, Indianapolis, In). The mAbs were purified on protein G columns (GE Healthcare Biosciences, Pittsburg, PA) and characterized by SDS-PAGE and Western blotting using anti-mouse-IgG1-HRP (Southern Biotech, Birmingham, AL). Binding of the produced mAb to the cognate receptor was verified by FACS on CHO cells stably transfected with mouse DEC, using PE-conjugated goat anti-mouse-IgG (Jackson ImmunoResearch, West Grove, PA). For expression of soluble LmSTI1a protein, the DNA encoding the first 1194 nucleotides of the LmSTI1 gene was amplified by PCR using primers described in Table S2. The PCR products were digested with BamHI and NotI, gel purified, and ligated in frame into the pET28b-SMT3 vector (generous gift from Dr. E. Mossessova, Memorial Sloan-Kettering Cancer Center) [61], followed by transformation into BL21 *Escherichia coli* (Life Technologies). Soluble protein was purified by nickel–agarose chromatography (Qiagen, Valencia, CA), followed by removal of His10-Smt3 with protease Ulp1, as described previously [61]. The concentration of all proteins produced, i.e., soluble protein and mAbs, was determined using the DC protein assay kit (BioRad, Hercules, CA). All proteins were examined for the presence of endotoxin using the Limulus amebocyte lysate assay, QCL-1000 (Cambrex, Walkersville, MD), and if necessary, removed using Triton-X, as described previously [62].

### Parasite Growth and Preparation of SLA

*L. major* (Friedlin strain, generously provided by Frederick S. Buckner, University of Washington) promastigotes were cultured at 27°C in M199 medium (Life Technologies) supplemented with 20% heat-inactivated FCS (Life Technologies), 0.5% penicillin/streptomycin (Life Technologies), 0.1 mM adenine (in 1 N NaOH), and 5 µg/ml hemin (in 50% triethanolamine) (both from Sigma) (Complete M199). In all experiments, promastigotes were used after one to three passages *in vitro*. Infective-stage metacyclic promastigotes were isolated from stationary cultures (4–5 days old) by negative selection of noninfective forms using peanut agglutinin (PNA, Vector Laboratories, Inc., Burlingame, CA. [63]). SLA was prepared from log-phase promastigotes (approximately 10^8^ parasites/ml), washed three times in PBS, and lyzed by seven cycles of freezing and thawing. Protein concentration was assessed by the BCA protein assay kit (Thermo Scientific, Rockford, Il).

### Mice Immunization and *in vivo* Challenge with *L. major*

The mice were immunized intraperitoneally one time with fusion mAbs in the presence of a stimulus for DC maturation, which was 50 µg poly ICLC (Oncovir, Washington, DC) together with 25 µg IC10 agonistic anti-CD40 mAb (produced in house). For parasite challenge experiments, anti-CD40 was omitted and the mice were inoculated subcutaneously in the footpad (50 µl per pad) in a prime-boost regimen consisting of two doses of 1–10 µg of fusion mAbs in the presence of 50 µg poly ICLC administered 1 month apart. Two to twelve weeks after the last vaccination, the animals were completely anesthetized with 1–1.5 mg Nembutal sodium (OVATION Pharmaceuticals, Inc.) and challenged with *L. major* stationary-phase metacyclic parasites. For high-dose challenge experiments, the mice were subcutaneously infected with 1×10^6^ parasites in the contralateral footpad to the vaccination site, and disease progression was monitored by measurement of footpad swelling with a caliper. In the low-dose challenge experiments, the mice were intradermally infected with 200–1000 parasites (in 20 µl) in the ear pinna. The animals were sacrificed when signs of footpad or ear ulceration became apparent at approximately 8–14 weeks after infection.

### Estimation of Parasite Loads

The amount of viable parasites in the infected ear, footpad, and draining LN were determined by a limiting dilution assay, as described previously [64]. In brief, infected ears and footpads were collected, weighed, and homogenized individually in 5 ml of complete M199. Single-cell suspensions were prepared from ear-infected draining LN in complete M199 and adjusted to equal cell numbers and volumes. In all cases, 10-fold serial dilutions were performed, and for each dilution, 12 replicates were plated on 96-well round-bottom plates, which were incubated for 10–15 days at 27°C in a CO_2_-free incubator. The number of parasites was estimated by multiplying the reciprocal of the last dilution showing at least one positive well with the initial dilution factor. Parasite loads were expressed as the number of *L. major* parasites per homogenized infected ear or footpad or per total cells in the draining LN.

### Intracellular Cytokine Staining and *in vitro* Proliferation Assay

Spleens were force-passed through a 70-µm cell strainer to obtain a homogeneous cell suspension in RPMI 1640 medium (Life Technologies) complemented with 10% FBS, 10 U/ml penicillin/streptomycin (Life Technologies), 200 µM/ml glutamine (Life Technologies), and 50 µM 2-mercaptoethanol (Sigma-Aldrich, St. Louis, MO). Red blood cells were lysed by incubating with RBC lysis buffer (BioWhittaker, Walkersville, MD) for 1 min. Bulk splenocytes were restimulated with 15-mer peptide mix from LmSTI1 (2 µg/ml) or nonreactive LeIF (2 µg/ml) in the presence of 2 µg/ml of costimulatory anti-CD28 (clone 37.51, ATCC produced in house) for 6 h at 37°C, adding 10 µg/ml Brefeldin A (BFA; 10 µg/ml, Sigma-Aldrich) for the last 5 h of incubation in order to allow accumulation of intracellular cytokines. In some experiments, the splenocytes were labelled with CFSE to evaluate the proliferative capacity of primed T cells [22], [24]. In brief, bulk splenocytes (1×10^7^ cells/ml) were labeled with 2.5 µM CFSE at 37°C for 10 min. CFSE-labeled T cells were then restimulated with 15-mer peptide mix from LmSTI1 (0.2 µg/ml) or nonreactive LeIF (0.2 µg/ml) for 4 days, followed by restimulation with 2 µg/ml reactive peptide mix for another 6 h in the presence of BFA for evaluation of intracellular cytokines. The cells were washed, incubated for 10 min at 4°C with anti-CD16/32 mAb (2.4G2, produced in house) to block Fcγ receptors, and stained with mAbs against surface molecules for 20 min at 4°C. The cells were then fixed, permeabilized (Cytofix/Cytoperm; BD Biosciences), and stained with mAbs against cytokines. 1–3×10^5^ live CD3^+^ cells were acquired on a BD LSR II Flow cytometer (BD Biosciences), and data were analyzed with FlowJo Software (TreeStar, San Carlos, CA).

### Cytokine ELISAs

Single-cell suspensions were prepared from ear-infected draining LN. 5×10^5^ cells/well (96-well round-bottom plates) were plated in the presence of 10 µg/ml SLA. Seventy-two hours later, the culture supernatant was harvested and IFN-γIL-4, and IL-10 production was evaluated by ELISA (eBioscience), following the manufacturer’s instructions.

### Statistical Analysis

Data were analyzed and charts were generated using Prism 5 GraphPad software (San Diego, CA). Comparisons between two groups were performed by unpaired Student’s t-test. Comparisons between three or more groups were performed by one-way ANOVA analysis (multiple comparisons Tukey’s post hoc test). P values ≤0.05 were considered statistically different and labeled with a single asterisk (*), in contrast to P values of ≤0.01 (**) or ≤0.001 (***).

## Supporting Information

Figure S1 **STI1 aa sequence alignment from** ***L. major*** **,** ***L. infantum*** **,** ***L. donovani*** **, and** ***L. braziliensis*** **.** Amino acid sequences were predicted from cDNA sequences obtained from NCBI database (www.ncbi.nlm.nih.gov). Residues matching between different *Leishmania* species are shown in dark gray boxes.
(TIF)

Figure S2 **Quality control of anti-DEC mAb engineering to expressed distinct** ***L. major*** **antigens.** (A) STI1 from *L. major* was divided into two fragments: the N-terminal portion (aa 1–398, red, LmSTI1a) and the C-terminal portion (aa 401–546, blue, LmSTI1b). Each fragment was cloned in frame into the C-terminal domain of the heavy chain of anti-DEC mAb or a control Ig mAb without receptor affinity (represented in B, left diagram). Furthermore, a small peptide (aa 344–366, pink) was cloned in frame to the C-terminal domain of anti-DEC mAb after a short linker (represented in B, right diagram). (B) Diagrammatic representation of anti-DEC mAb conjugated with LmSTI1a and LmSTI1b (left diagram) or with LmSTI1_344–366_ (right diagram). (C) Coomassie blue-stained 10% (vol/vol) SDS-PAGE reducing gel comparing fusion mAbs with the molecular mass in kDa. (D) Western blotting of fusion mAbs using HRP-conjugated anti-mouse IgG1. Molecular mass is indicated in kDa. (E) Binding of the fusion mAbs to their cognate receptor analyzed by FACS. CHO cells transfected to expressed mouse DEC (red) or control non-transfected CHO cells (CHO-NEO, blue) were incubated with graded doses (0.02–2 µg) of fusion mAb, followed by staining with PE-labeled anti-mouse IgG. (F) The N-terminal portion of LeIF (aa 1–226) was cloned in frame into the C-terminal domain of anti-DEC mAb or a control Ig mAb. (G) Panel shows Coomassie blue-stained SDS-PAGE as in C. (H) Panel shows Western blotting as explained in D. (I) FACS plots show binding to CHO cells expressing DEC as explained in E.
(TIF)

Figure S3 **Multifunctional CD4^+^ T cell responses are elicited by anti-DEC-LmSTI1a in Balb/c mice.** Balb/c mice were intraperitoneally immunized with 1 µg of anti-DEC-LmSTI1a or control Ig-LmSTI1a mAbs in the presence of 50 µg poly ICLC and 25 µg anti-CD40. Fourteen days later, splenocytes were restimulated *in vitro* with a reactive LmSTI1a peptide mix in the presence of BFA for 6 h. The production of IFN-γ, TNF-α and IL-2 was evaluated by FACS after intracellular cytokine staining, and the frequencies of CD4^+^ IFN-γ^+^ T cells also producing TNF-α and/or IL-2 are shown as the mean ± SEM (n = 6).
(TIF)

Figure S4 **Identification of LmSTI1a-, LeIF-, and LmSTI1b-CD4^+^ T cell epitopes in Balb/c and C57BL/6 mice.** Balb/c (A) or C57BL/6 (B) mice were immunized with anti-DEC-LmSTI1a mAb in the presence of 50 µg poly ICLC and 25 µg anti-CD40 mAb. Two weeks later, splenocytes were restimulated with 2 µg/ml of the indicated individual LmSTI1a peptide from pools 1, 2, and 8. IFN-γ production was evaluated by flow cytometry after intracellular cytokine staining, and the bars are shown as the mean ± SEM (n = 3). The aa sequence of the reactive peptides is shown. The sequences of the previously described reactive epitopes in Balb/c mice are underlined [37]. (C) Splenocytes from Balb/c mice immunized 14 days previously with anti-DEC-LeIF mAb plus adjuvant were restimulated with 2 µg/ml of the indicated individual peptides from pool 5. IFN-γ production was evaluated by flow cytometry after intracellular cytokine staining. Bars are shown as the mean ± SEM (n = 3), and the aa sequence of the reactive peptide is shown. (D) As in C, but animals were immunized with anti-DEC-LmSTI1b plus adjuvant. The sequence of a previously described reactive epitope in Balb/c mice is underlined [37].
(TIF)

Figure S5 **Delivery of LmsTI1a to DCs using anti-DEC mAbs protects mice against cutaneous leishmaniasis.** (A) Balb/c mice were primed and boosted 1 month apart with 10 µg of anti-DEC mAbs coupled with LACK, LeIF, or LmSTI1a, or a control Ig-LmSTI1a mAbs in the presence of 50 µg of poly ICLC. Ten to 15 days after the last immunization, the mice were challenged with a single dose of 200–1000 *L. major* metacyclic promastigotes. Representative lesions in the ears of Balb/c mice 12 weeks after challenge are shown. (B) Balb/c mice were vaccinated in a prime-boost regimen consisting of two doses of 10 µg of anti-DEC or control Ig mAbs conjugated with either LmSTI1a, LmSTIb, or LeIF, subcutaneously administered in the presence of poly ICLC (50 µg) in the right footpad. Two weeks after the boost, the mice were subcutaneously challenged in the left footpad with 1–2×10^6^ *L. major* metacyclic promastigotes. Vaccine efficacy was determined by weekly measurement of the thickness of the infected footpad. The mean ± SEM is shown (n ≥4). (C) As in B, but the number of parasites in the infected footpad is shown as the mean ± SEM (n ≥4). n.d. = not detected. (D) As in A, but C57BL/6 mice were primed and boosted 1 month apart with two subcutaneous doses of anti-DEC-LmSTI1a (1 µg) in the presence of 50 µg of poly ICLC. Ten to 15 days after the last immunization, mice were challenged with a single dose of 200–1000 *L. major* metacyclic promastigotes. The quantification of parasites obtained from the infected ears was determined 4 weeks after challenge. The mean ± SEM is shown (n = 4–5).
(TIF)

Figure S6 **Cytokine profile from ear-infected draining LN cells stimulated** ***in vitro*** **with SLA after intradermal challenge with** ***L. major*** **.** Total cell suspensions of ear-infected draining LN obtained from mice immunized with different preparations, as described in Figure 6, were restimulated *in vitro* for 72 h with 10 µg/ml SLA. IL-4 (A) and IFN-γ(B) levels in the supernatants of the cultures were determined by ELISA. The cultures were set in triplicates, and the data shows the mean ± SEM (n ≥4).
(TIF)

Table S1 **Peptide sequences covered by the immunogenic pools.**
(DOC)

Table S2 **Primers sequences.**
(DOC)
