# Correction: Targeting *Leishmania major* Antigens to Dendritic Cells In Vivo Induces Protective Immunity

**DOI:** 10.1371/annotation/5149bf8e-3843-4865-a726-0ca2820ee8f8

**Published:** 2013-09-19

**Authors:** Ines Matos, Olga Mizenina, Ashira Lubkin, Ralph M. Steinman, Juliana Idoyaga

Multiple funding organizations and grants were incorrectly omitted from the Funding Statement. The Funding Statement should read: "Funding was provided by NIH/NIAID grant AI13013 (to R.M.S.), The Rockefeller University Center for Clinical and Translational Science Grant Award Number ULTR000043 (to J.I.), NIH/NIAMS grant 1K99AR062595 (to J.I.), and by the Fundação para a Ciência e Tecnologia PhD scholarship SFRH/BD/41073/2007 (to I.M.). The funders had no role in study design, data collection and analysis, decision to publish, or preparation of the manuscript." 

