# Supplementary figures and images for: Targeting Leishmania major Antigens to Dendritic Cells In Vivo Induces Protective Immunity
Source: PLoS One. 2013 Jun 26;8(6):e67453. doi: 10.1371/journal.pone.0067453 (PMC3694010; doi:10.1371/journal.pone.0067453)

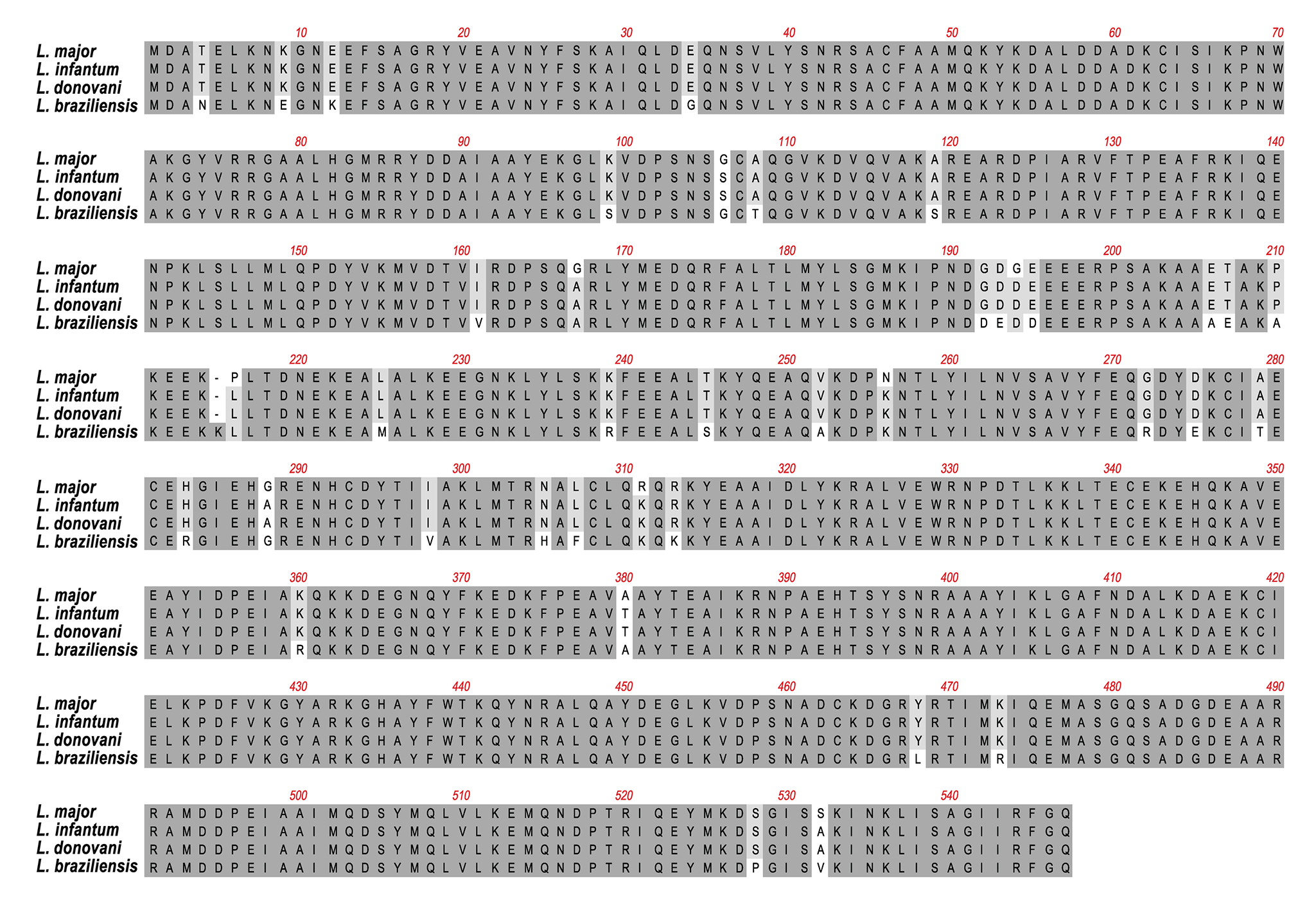

Supplement: Figure S1 — STI1 aa sequence alignment from L. major , L. infantum , L. donovani , and L. braziliensis . Amino acid sequences were predicted from cDNA sequences obtained from NCBI database (www.ncbi.nlm.nih.gov). Residues matching between different Leishmania species are shown in dark gray boxes. (TIF) [file pone.0067453.s001.tif]

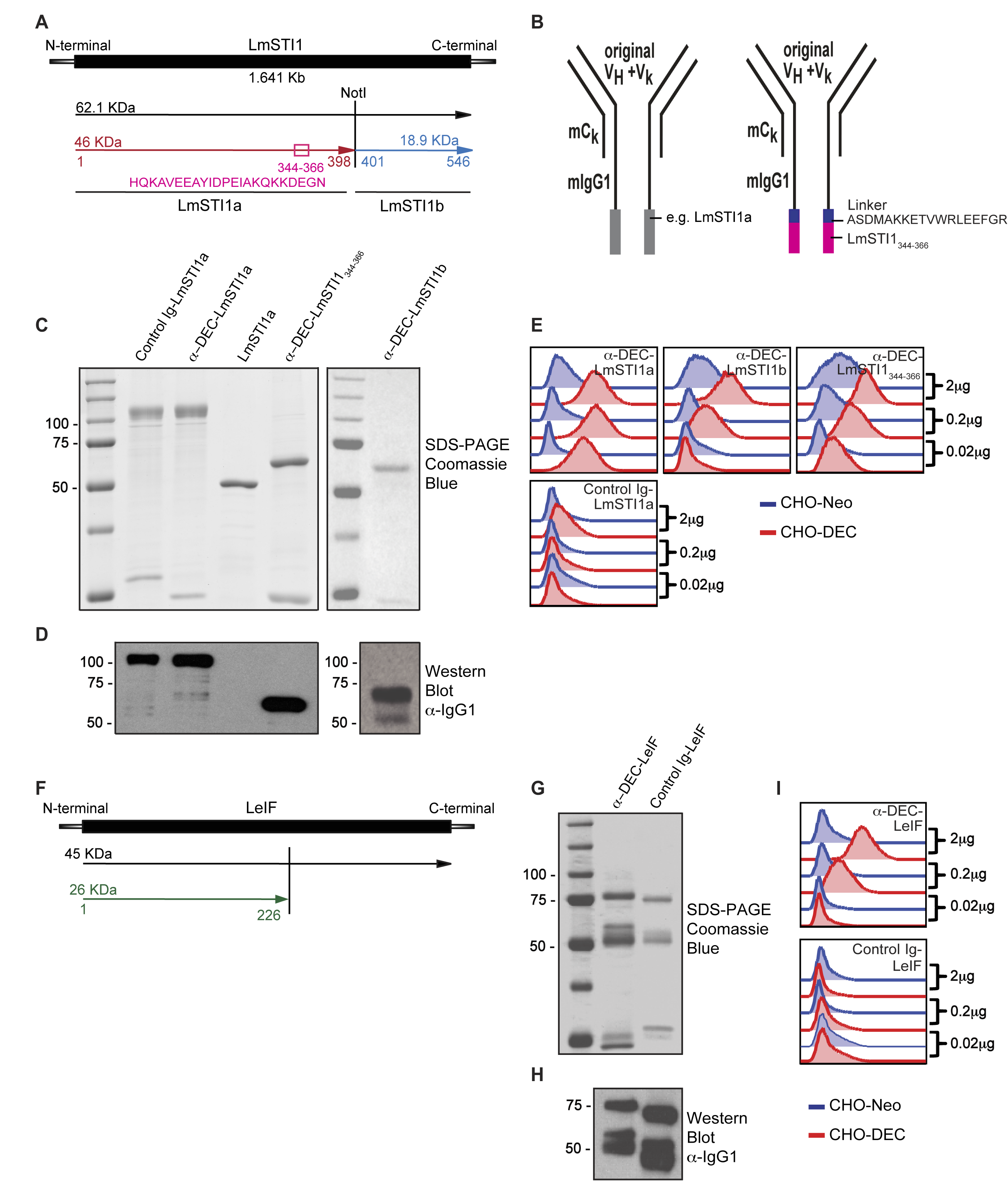

Supplement: Figure S2 — Quality control of anti-DEC mAb engineering to expressed distinct L. major antigens. (A) STI1 from L. major was divided into two fragments: the N-terminal portion (aa 1–398, red, LmSTI1a) and the C-terminal portion (aa 401–546, blue, LmSTI1b). Each fragment was cloned in frame into the C-terminal domain of the heavy chain of anti-DEC mAb or a control Ig mAb without receptor affinity (represented in B, left diagram). Furthermore, a small peptide (aa 344–366, pink) was cloned in frame to the C-terminal domain of anti-DEC mAb after a short linker (represented in B, right diagram). (B) Diagrammatic representation of anti-DEC mAb conjugated with LmSTI1a and LmSTI1b (left diagram) or with LmSTI1344–366 (right diagram). (C) Coomassie blue-stained 10% (vol/vol) SDS-PAGE reducing gel comparing fusion mAbs with the molecular mass in kDa. (D) Western blotting of fusion mAbs using HRP-conjugated anti-mouse IgG1. Molecular mass is indicated in kDa. (E) Binding of the fusion mAbs to their cognate receptor analyzed by FACS. CHO cells transfected to expressed mouse DEC (red) or control non-transfected CHO cells (CHO-NEO, blue) were incubated with graded doses (0.02–2 µg) of fusion mAb, followed by staining with PE-labeled anti-mouse IgG. (F) The N-terminal portion of LeIF (aa 1–226) was cloned in frame into the C-terminal domain of anti-DEC mAb or a control Ig mAb. (G) Panel shows Coomassie blue-stained SDS-PAGE as in C. (H) Panel shows Western blotting as explained in D. (I) FACS plots show binding to CHO cells expressing DEC as explained in E. (TIF) [file pone.0067453.s002.tif]

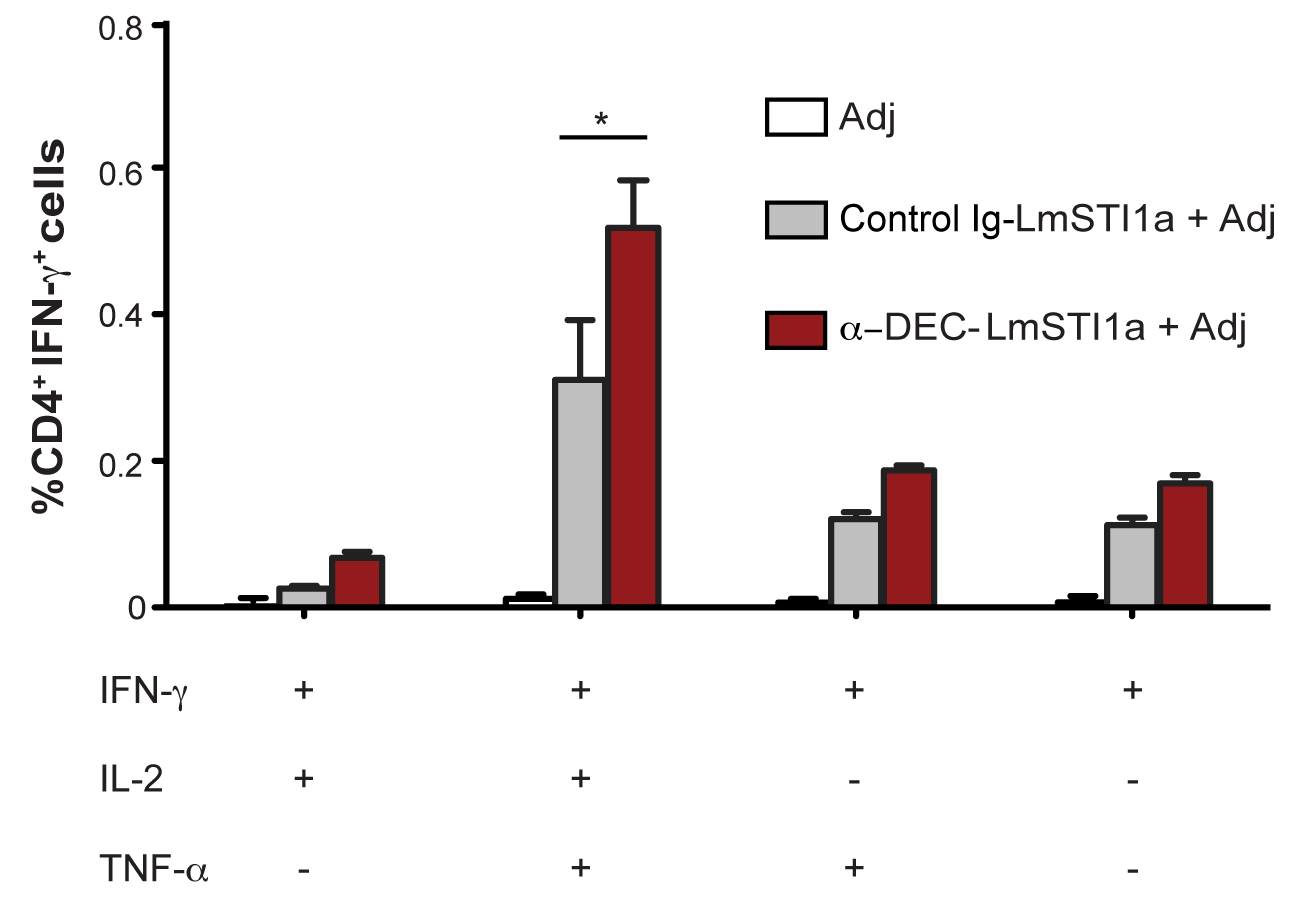

Supplement: Figure S3 — Multifunctional CD4+ T cell responses are elicited by anti-DEC-LmSTI1a in Balb/c mice. Balb/c mice were intraperitoneally immunized with 1 µg of anti-DEC-LmSTI1a or control Ig-LmSTI1a mAbs in the presence of 50 µg poly ICLC and 25 µg anti-CD40. Fourteen days later, splenocytes were restimulated in vitro with a reactive LmSTI1a peptide mix in the presence of BFA for 6 h. The production of IFN-γ, TNF-α and IL-2 was evaluated by FACS after intracellular cytokine staining, and the frequencies of CD4+ IFN-γ+ T cells also producing TNF-α and/or IL-2 are shown as the mean ± SEM (n = 6). (TIF) [file pone.0067453.s003.tif]

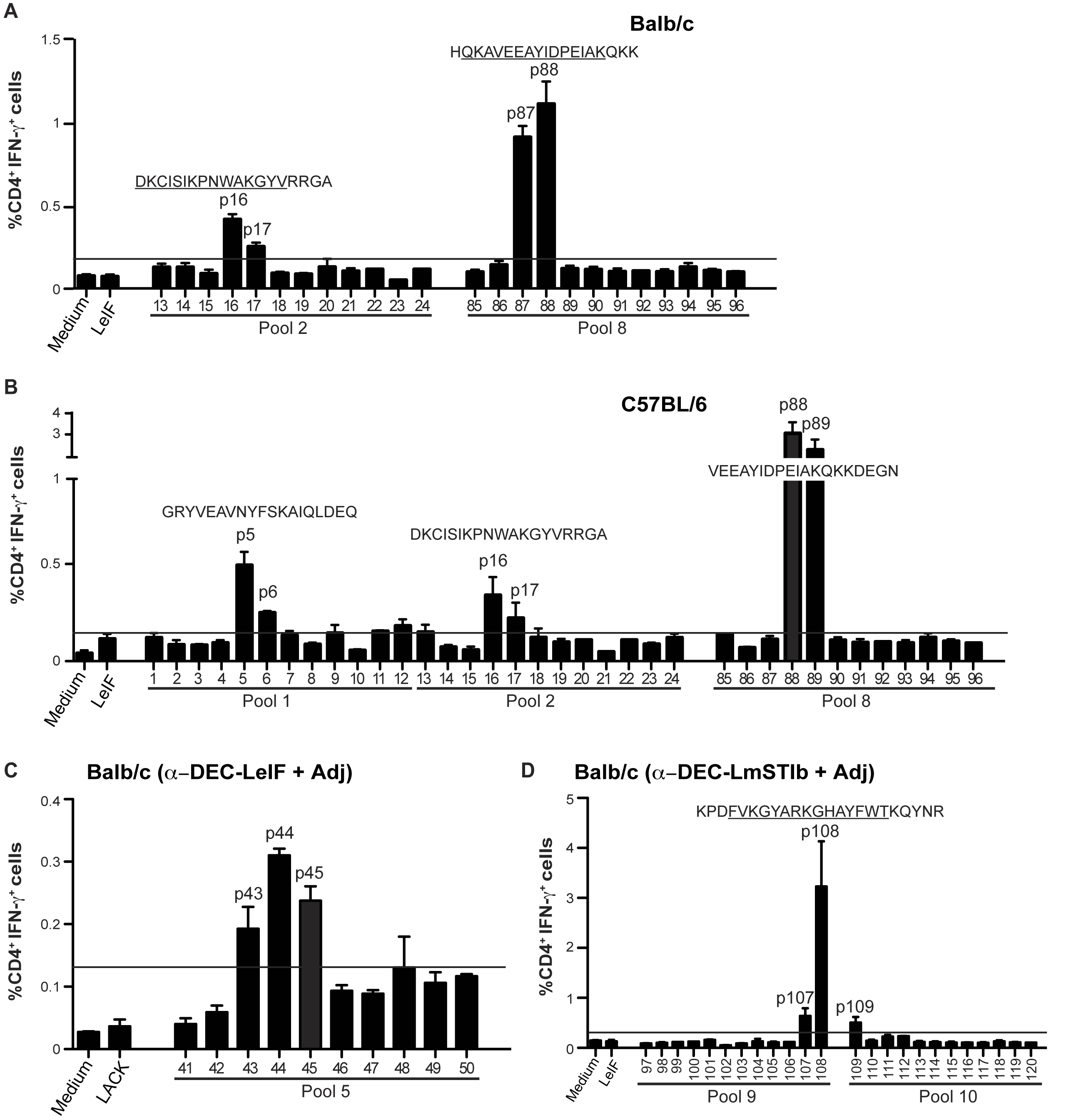

Supplement: Figure S4 — Identification of LmSTI1a-, LeIF-, and LmSTI1b-CD4+ T cell epitopes in Balb/c and C57BL/6 mice. Balb/c (A) or C57BL/6 (B) mice were immunized with anti-DEC-LmSTI1a mAb in the presence of 50 µg poly ICLC and 25 µg anti-CD40 mAb. Two weeks later, splenocytes were restimulated with 2 µg/ml of the indicated individual LmSTI1a peptide from pools 1, 2, and 8. IFN-γ production was evaluated by flow cytometry after intracellular cytokine staining, and the bars are shown as the mean ± SEM (n = 3). The aa sequence of the reactive peptides is shown. The sequences of the previously described reactive epitopes in Balb/c mice are underlined [37]. (C) Splenocytes from Balb/c mice immunized 14 days previously with anti-DEC-LeIF mAb plus adjuvant were restimulated with 2 µg/ml of the indicated individual peptides from pool 5. IFN-γ production was evaluated by flow cytometry after intracellular cytokine staining. Bars are shown as the mean ± SEM (n = 3), and the aa sequence of the reactive peptide is shown. (D) As in C, but animals were immunized with anti-DEC-LmSTI1b plus adjuvant. The sequence of a previously described reactive epitope in Balb/c mice is underlined [37]. (TIF) [file pone.0067453.s004.tif]

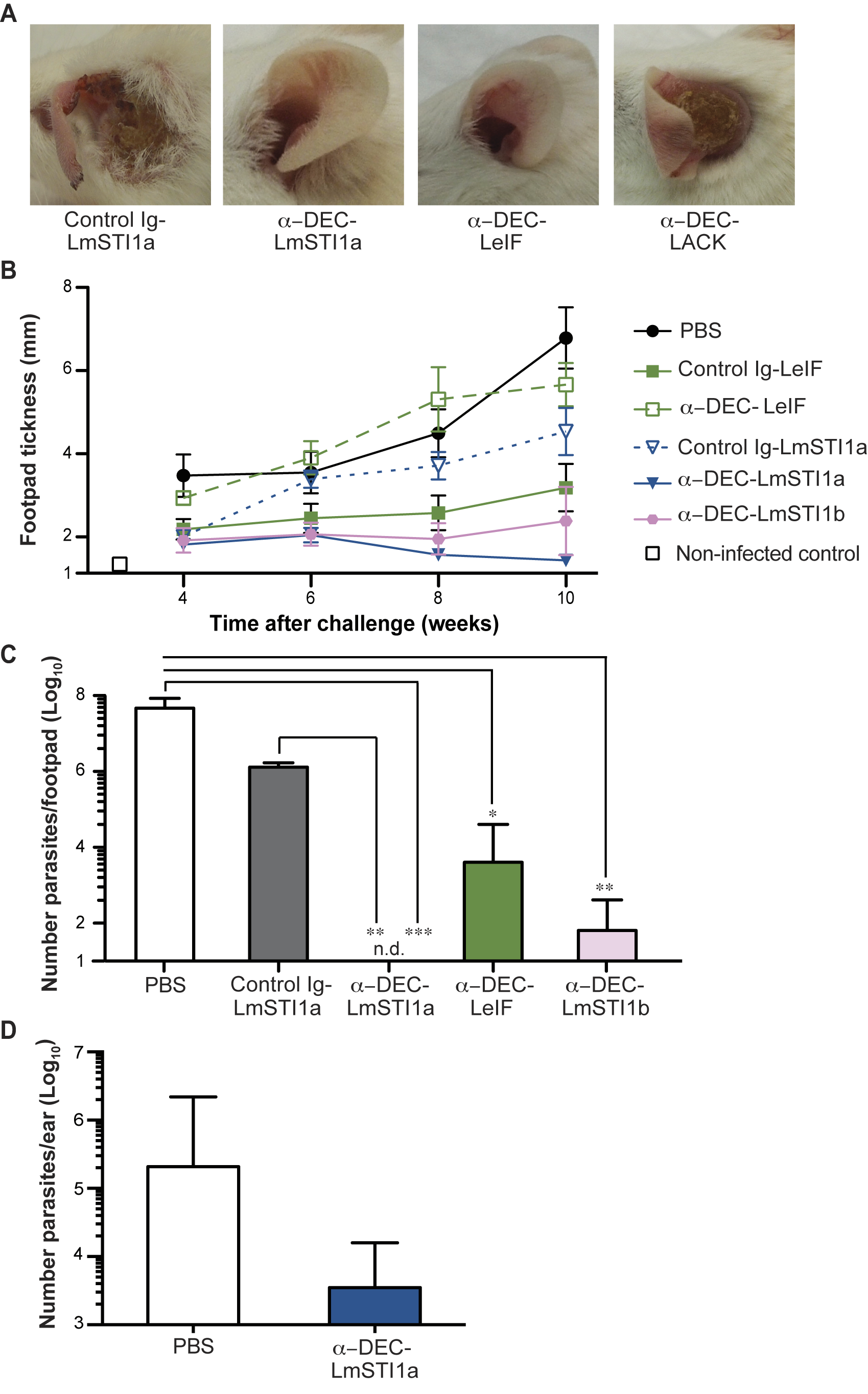

Supplement: Figure S5 — Delivery of LmsTI1a to DCs using anti-DEC mAbs protects mice against cutaneous leishmaniasis. (A) Balb/c mice were primed and boosted 1 month apart with 10 µg of anti-DEC mAbs coupled with LACK, LeIF, or LmSTI1a, or a control Ig-LmSTI1a mAbs in the presence of 50 µg of poly ICLC. Ten to 15 days after the last immunization, the mice were challenged with a single dose of 200–1000 L. major metacyclic promastigotes. Representative lesions in the ears of Balb/c mice 12 weeks after challenge are shown. (B) Balb/c mice were vaccinated in a prime-boost regimen consisting of two doses of 10 µg of anti-DEC or control Ig mAbs conjugated with either LmSTI1a, LmSTIb, or LeIF, subcutaneously administered in the presence of poly ICLC (50 µg) in the right footpad. Two weeks after the boost, the mice were subcutaneously challenged in the left footpad with 1–2×10^6^ L. major metacyclic promastigotes. Vaccine efficacy was determined by weekly measurement of the thickness of the infected footpad. The mean ± SEM is shown (n ≥4). (C) As in B, but the number of parasites in the infected footpad is shown as the mean ± SEM (n ≥4). n.d. = not detected. (D) As in A, but C57BL/6 mice were primed and boosted 1 month apart with two subcutaneous doses of anti-DEC-LmSTI1a (1 µg) in the presence of 50 µg of poly ICLC. Ten to 15 days after the last immunization, mice were challenged with a single dose of 200–1000 L. major metacyclic promastigotes. The quantification of parasites obtained from the infected ears was determined 4 weeks after challenge. The mean ± SEM is shown (n = 4–5). (TIF) [file pone.0067453.s005.tif]

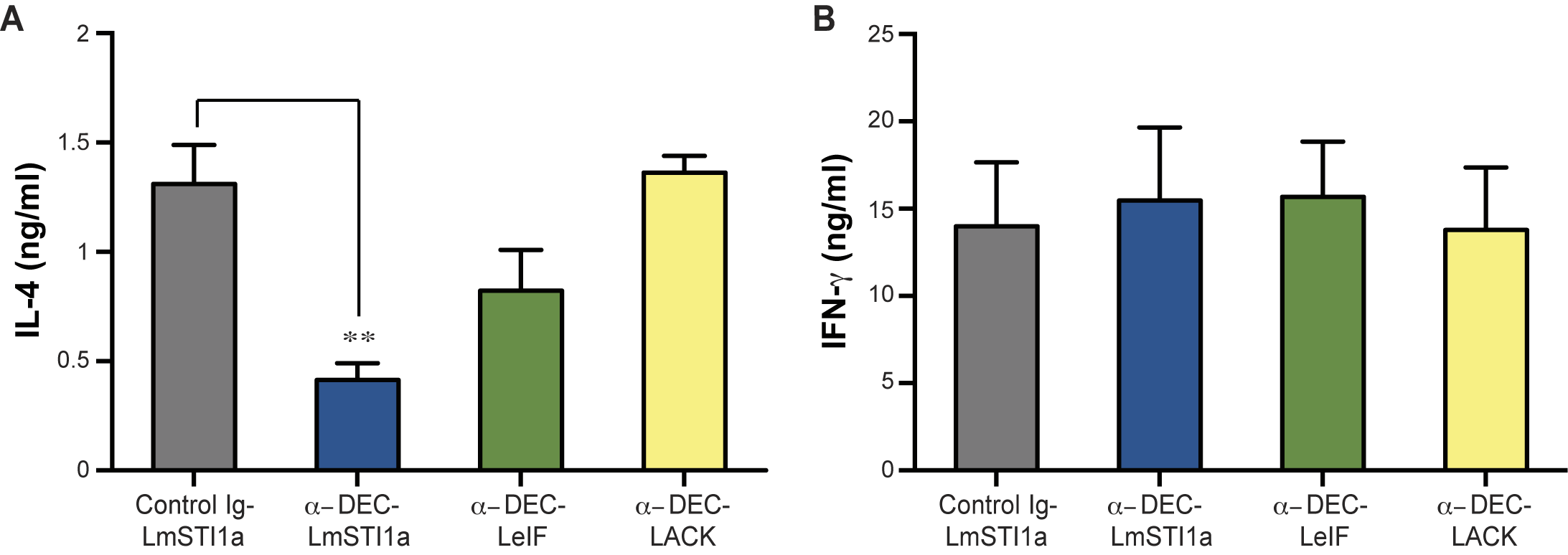

Supplement: Figure S6 — Cytokine profile from ear-infected draining LN cells stimulated in vitro with SLA after intradermal challenge with L. major . Total cell suspensions of ear-infected draining LN obtained from mice immunized with different preparations, as described in Figure 6, were restimulated in vitro for 72 h with 10 µg/ml SLA. IL-4 (A) and IFN-γ(B) levels in the supernatants of the cultures were determined by ELISA. The cultures were set in triplicates, and the data shows the mean ± SEM (n ≥4). (TIF) [file pone.0067453.s006.tif]
