# Supplementary material for: Targeting Leishmania major Antigens to Dendritic Cells In Vivo Induces Protective Immunity
Source: PLoS One. 2013 Jun 26;8(6):e67453. doi: 10.1371/journal.pone.0067453 (PMC3694010; doi:10.1371/journal.pone.0067453)
Supplement: Table S1 — Peptide sequences covered by the immunogenic pools. (DOC) [file pone.0067453.s007.doc]

Table S1: Peptide sequences covered by the immunogenic pools

| **Strain** | **MHCII Haplotype** | **LmSTI peptide pool** | **Position (aa)** | **Sequence** |
| --- | --- | --- | --- | --- |
| C57BL/6 | H-2^b^ | 1 | 1–59 | MDATELKNKGNEEFSAGRYVEAVNYFSKAIQLDEQNSVLYSNRSACFAAMQKYKDALDD |
| C57BL/6 and Balb/c | H-2^b^  H-2^d^ | 2 | 49–108 | AMQKYKDALDDADKCISIKPNWAKGYVRRGAALHGMRRYDDAIAAYEKGLKVDPSNSGCA |
| C57BL/6 and Balb/c | H-2^b^  H-2^d^ | 8 | 335–392 | KLTECEKEHQKAVEEAYIDPEIAKQKKDEGNQYFKEDKFPEAVAAYTEAIKRNPAEH |
