# Supplementary material for: Targeting Leishmania major Antigens to Dendritic Cells In Vivo Induces Protective Immunity
Source: PLoS One. 2013 Jun 26;8(6):e67453. doi: 10.1371/journal.pone.0067453 (PMC3694010; doi:10.1371/journal.pone.0067453)
Supplement: Table S2 — Primers sequences. (DOC) [file pone.0067453.s008.doc]

Table S2: Primers sequences

| **Target region** | **Primer Sequence (5′–3′)** | **Construct name** | **Genbank #** |
| --- | --- | --- | --- |
| N-terminus 398 aa residues from LmSTI ORF | *Forward* – CG GCT AGC ATG GAC GCA ACT GAG CTG AAG AAC A | Mouse anti-DEC-LmSTIa or Control Ig-LmSTIa | XM_001681088.1 |
|  | *Reverse* – TAC GCG GCC GCT CAC TAG CGA TTG CTG TAG GA |  |  |
|  | *Forward* – CG GGA TCC ATG GAC GCA ACT GAG CTG AAG AAC A | Soluble LmSTIa |  |
|  | *Reverse* – TAC GCG GCC GCT CAC TAG CGA TTG CTG TAG GA |  |  |
| C-terminus 146 aa residues from LmSTI ORF | *Forward* – CG GCT AGC TAC ATC AAG CTT GGA GCC TTC A | Mouse anti-DEC-LmSTIb |  |
|  | *Reverse –* TAA AGC GGC CGC TCA CTA CTG ACC AAA ACG AAT GAT GCC AGC |  |  |
| 344–366 aa residues from LmSTI ORF | *Forward peptide* – CAC CAA AAG GCG GTG GAG GAA GCC TAC ATC GAT CCT GAG ATC GCG AAG CAG AAG AAA GAC GAA GGT AAC TGA GC | Mouse anti-DEC-LmSTI 344–366 |  |
|  | *Reverse peptide* – GGC CGC TCA GTT ACC TTC GTC TTT CTT CTG CTT CGC GAT CTC AGG ATC GAT GTA GGC TTC CTC CAC CGC CTT TTG GTG CCT ACC GA |  |  |
|  | *Forward linker* – CT AGC GAC ATG GCC AAG AAG GAG ACA GTC TGG AGG CTC GAG GAG TTC GGT AGG |  |  |
|  | *Reverse linker* – A CTC CTC GAG CCT CCA GAC TGT CTC CTT CTT GGC CAT GTC G |  |  |
| N-terminus 266 aa residues from LeIF ORF | *Forward* – CG GCT AGC ATG GCG CAG AAT GAT AAG ATC GCC C | Mouse anti-DEC-LeIF or Control Ig-LeIF | XM_001687418.1 |
|  | *Reverse* *–* TAA AGC GGC CGC TCA CTA GTC GCG CAT GAA CTT CTT CGT C |  |  |

Underlined sequences are the restriction enzyme sites used for cloning
